# Identification of Clinically Relevant HIV Vif Protein Motif Mutations through Machine Learning and Undersampling

**DOI:** 10.3390/cells12050772

**Published:** 2023-02-28

**Authors:** José Salomón Altamirano-Flores, Luis Ángel Alvarado-Hernández, Juan Carlos Cuevas-Tello, Peter Tino, Sandra E. Guerra-Palomares, Christian A. Garcia-Sepulveda

**Affiliations:** 1 Engineering Faculty, UASLP, San Luis Potosí 78290, Mexico; 2 School of Computer Science, University of Birmingham, Birmingham B15 2TT, UK; 3 Viral and Human Genomics Laboratory, Faculty of Medicine, UASLP, San Luis Potosí 78210, Mexico

**Keywords:** HIV-Vif, undersampling, machine learning

## Abstract

Human Immunodeficiency virus (HIV) and its clinical entity, the Acquired Immunodeficiency Syndrome (AIDS) continue to represent an important health burden worldwide. Although great advances have been made towards determining the way viral genetic diversity affects clinical outcome, genetic association studies have been hindered by the complexity of their interactions with the human host. This study provides an innovative approach for the identification and analysis of epidemiological associations between HIV Viral Infectivity Factor (Vif) protein mutations and four clinical endpoints (Viral load and CD4 T cell numbers at time of both clinical debut and on historical follow-up of patients. Furthermore, this study highlights an alternative approach to the analysis of imbalanced datasets, where patients without specific mutations outnumber those with mutations. Imbalanced datasets are still a challenge hindering the development of classification algorithms through machine learning. This research deals with Decision Trees, Naïve Bayes (NB), Support Vector Machines (SVMs), and Artificial Neural Networks (ANNs). This paper proposes a new methodology considering an undersampling approach to deal with imbalanced datasets and introduces two novel and differing approaches (MAREV-1 and MAREV-2). As theses approaches do not involve human pre-determined and hypothesis-driven combinations of motifs having functional or clinical relevance, they provide a unique opportunity to discover novel complex motif combinations of interest. Moreover, the motif combinations found can be analyzed through traditional statistical approaches avoiding statistical corrections for multiple tests.

## 1. Introduction

Human immunodeficiency virus (HIV) and its clinical entity, the Acquired Immunodeficiency Syndrome (AIDS) continue to represent an important health burden worldwide. Since the first reports of HIV more than 35 years ago, 78 million people have been infected with HIV and 35 million have died from AIDS-related illnesses. In 2021, approximately 1.5 million people contracted HIV and 650,000 people died from HIV-related diseases (UNAIDS, https://www.unaids.org/en, accessed on 28 October 2022. Although the overall number of new infections has declined since 2010, the resource limited countries of Latin America, Asia, and Africa have shown a steady increase in new infections and excess deaths due to HIV [1]. Different strategies have been employed in the fight against HIV and AIDS, mostly focused on either preventative measures or the development of novel anti-retroviral drugs targeting the main viral enzymes involved in HIV replication [2]. On the other hand, current HIV research efforts continue to focus on increasing our understanding of viral-host interactions at the molecular level, with the aim to discover those worth exploiting to interfere with viral tropism, fusion, replication, integration, and transmission.

Our understanding of the function of some viral proteins such as the protease, reverse transcriptase, and integrase enzymes has allowed for the development of potent preventative and therapeutic strategies [3]. However, for some accessory and non-structural viral proteins, little is known with regards to the function and their potential as candidate targets for antiviral drug development. While the use of molecular biology techniques allows for an estimation of functional or clinical relevance of these proteins, complex genetic and clinical variable comparisons decrease the statistical power of such studies.

The HIV genome has 9719 base pairs (HXB2 reference strain) and a total of 3 open reading frames encoded in a prototypical lentivirinae genome organization comprised of *gag*, *pol*, and *env* genes, long terminal repeat regions (LTRs) and accessory-protein-encoding regions (*Vif, vpr, tat, rev, vpu*, and *nef*). The *gag* gene encodes for the matrix, capsid, nucleocapsid, and p6 proteins, *pol* encodes for the enzymes protease, reverse-transcriptase, and integrase and *env* encodes for the glycoproteins GP41 and GP120. The different aforementioned accessory proteins facilitate or promote HIV replication and viral fitness. The best studied accessory proteins include *tat* (which acts as viral transcriptional transactivator), *rev* (which regulates RNA trafficking), and *nVifef* which promotes viral maturation and release from the host cell [4,5]. *Vif* is a 192-amino acid HIV accessory protein essential for replication. Vif protein counteracts human antiviral proteins of the APOlipoprotein Bmessenger RNA Editing enzyme, Catalytic polypeptide-like (APOBEC3) family. APOBEC3 proteins are zinc-dependent deaminases which mutate viral cytidine (dC) to uridine (dU) in both viral DNA and RNA molecules, thus interfering with the fidelity of the viral genome. APOBEC3 is a host innate mechanism that protects human cells from exogenous viruses and endogenous mobile retroelements. The Vif protein allows HIV to evade such innate mechanisms. This viral protein has recently become a candidate target for both therapeutic and preventive interventions in HIV/AIDS. Nevertheless, little is known about the clinical relevance of Vif accessory protein, particularly among HIV-infected patients of developing countries and Latin America [6].

Members of the human APOBEC family of proteins include APOBEC1, APOBEC2, APOBEC3, and the poorly expressed APOBEC4. The APOBEC3 subfamily has seven known members, including APOBEC3A, APOBEC3B, APOBEC3C, APOBEC3DE, APOBEC3F, APOBEC3G, and APOBEC3H. Among all APOBEC3 subfamily members, APOBEC3G is notable for exerting the strongest antiviral effect [7]. APOBEC3G is incorporated into the HIV-1 virions as they emerge from an infected cell when HIV-1 lacks the capacity to encode for Vif protein. During the second round of viral replication, after infecting a second cell, APOBEC3G would normally cause extensive dC to dU mutations of the single-stranded viral DNA during reverse transcription [8]. HIV’s Vif protein inhibits and interferes with APOBEC3G activity and thus renders the virus immune to this important innate immunity. However, HIV-1 evolution and quasi-species diversification within a single human being might lead to the accumulation of mutations in the Vif region, which might affect protein function and have clinical significance by either decreasing viral replication or affecting integration and transmission.

The use of machine learning approaches has been extensively applied to the search of statistical associations between genetic and clinical variables during the last years given their known capacity at tackling high dimensional data [9,10]. Previously, some research groups have applied combined algorithm based approaches, such as ANN coupled to genetic algorithms, grammatical evolution, and genetic programming, to the discovery of genetic associations and classification [11,12,13,14,15]. Other combined-algorithm approaches have been SVMs with genetic algorithms [16] and ANNs coupled to Rule Association Mining (Apriori algorithm) [17]. Although combining different machine learning approaches does not guarantee better performance, there is ample evidence supporting the statistical benefits and capabilities at discovering novel genetic associations in the context of infectious diseases [18,19].

One important factor in assessing the importance of different genetic variables mentioned in previously published studies is their combined effect on classification performance. We previously applied this approach to the study of HIV’s Vif gene mutations by using four different machine learning approaches for the discovery of clinical endpoint associations [20]. A mayor caveat to our previous effort was the availability of an imbalanced dataset arising from the difficulty in collecting large cohort samples and extensive genetic data. Data imbalance is a fundamental and challenging problem in machine learning that limits the power of small clinical datasets. This limitation has also been shown to be present in other non-medical applications such as fraud detection, finance, ecology, and biology [21,22]. As such, in this study we set forth to evaluating the performance of state-of-the-art machine learning approaches (Decision Trees, NB, SVMs, and ANNs) enhanced with an undersampling process for dealing with the data imbalance in the dataset. Furthermore, we present a probabilistic method capable of suggesting the most clinically relevant variable combinations associated to clinical outcomes.

The paper is organized as follows: Section 2 describes the dataset and the undersampling approach. The methods are presented in Section 3, followed by the results and conclusions sections.

## 2. Dataset

For the purpose of this study we relied on a previously consolidated dataset including Vif protein amino acid physicochemical changes and clinical outcome variables (CD4 T cell numbers and HIV viral load at both initial diagnosis and on follow-up) [23]. From the original 192 amino-acid sites conforming the Vif protein, those pertaining to 17 protein motifs were encoded into binary data as either conserved or mutated, as described previously [20]. Eight of the 17 variables representing Vif protein domains are known to interact with APOBEC3 proteins (herein designated as APOBEC-1 to APOBEC-8). Other motifs considered in this study include the Nuclear Localisation Inhibitory Signal (NLIS), two (CBFβ-1 and -2) interaction sites as well as three Cullin-5 binding sites (Cul5-1, Cul5-2, and Cul5-3). When the different Vif motif sequences implied a non-conservative change in physicochemical properties, the genetic variable for that motif was encoded as a “1”, and when the site was conserved it was encoded as “0”.

The values for the clinical endpoints (outcome class) were encoded based on thresholds recommended by the World Health Organization and the U.S. Centers for Disease Control and Prevention. The CD4Ini and CD4Hist clinical endpoints reflect the levels of CD4+ T cells number (cells/per micro liter) at the first time of diagnosis (CD4Ini) and as the median number of CD4+ cells from quarterly assessments during two years of patient follow-up (CD4Hist). For both CD4Ini and CD4Hist, ≥500 CD4+ T cells/μL corresponds to a value of “0”, as CD4+ T cell numbers above this threshold are not indicative of poor clinical prognosis. Contrarily, the clinical endpoint is encoded as “1”, when ≤500 CD4+ T cells/μL when the cell numbers are below normal and reflecting immunodeficiency. Similarly, VLIni and VLHist outputs reflect another clinical aspect used to assess HIV-prognosis, where high viral loads are associated with worsening clinical progression. As mentioned above, VLIni and VLHist reflect HIV viral titres at the time of initial diagnosis and the median of quarterly follow-up assessments of viral load (copies/milliliter). For both VLIni and VLHits ≥ 10,000 copies/mL/μL corresponds to a value of “1”, as viral loads above 10,000 cp/mL are suggestive of intense viral replication and worsening clinical prognosis. Contrarily, this value is encoded as “0”, when ≤500 copies/mL/μL when the viral load is below 10,000 cp/mL and stable [24].

### Undersampling

In the case of binary classification, the class-imbalance is defined as the over representation of one class (the majority class) over another class (the minority class). Over representation affects the learning process of the algorithms as most of them are designed to construct the most general and simplest hypothesis from the data [25]. Undersampling can lead to a bias towards the over-represented class during the learning process.

Different approaches have been used to resolve the problem of undersampling, which range from applying data balancing strategies (either undersampling or oversampling), modifying the machine learning process to address data imbalance or through data penalization to enhance minority class attribute detection [26]. Undersampling balancing strategies are the most popular approach as they are based on the original dataset, whereas oversampling requires the generation of artificial data, derived from the original dataset but not necessarily true in content [27].

As the use of oversampling involves the generation of artificial data, in this work we decided to use an undersampling approach to better preserve the biological distribution of genetic variables and clinical endpoints of our dataset.

**Figure 1:**
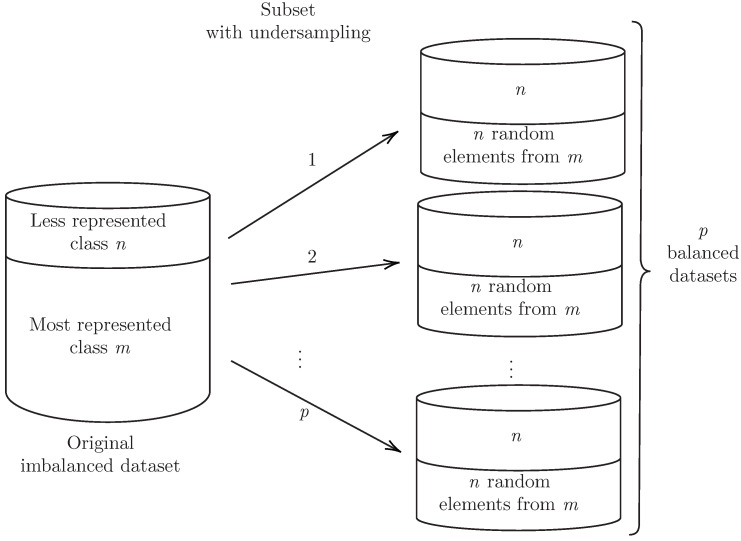
Producing multiple (*p*) balanced datasets through undersampling of the imbalanced dataset composed of a majority class (*m*) and a less represented, minority, class (*n*) by randomly removing majority class elements until m=n.

Figure 1 describes the undersampling process. The original dataset contains m+n examples where *n* is the minority class and *m* is the majority class. The algorithm identifies the least represented class (i.e., *n*) and then creates a new balanced dataset by subtracting *m* class elements until it is similar in size to *n* class subset. These undersampled balanced sets are generated 100 times (1,2,…,p), and each one is used for machine learning and training.

## 3. Methods

This paper compared the classification performance of the well-known machine learning methods: Decision Trees, NB, SVMs, and Multi-Layer Perceptron (MLP).

### 3.1. Decision Trees

Decision trees represent the simplest and most widely used non-parametric supervised learning method. There are many algorithmic implementations to generate decision trees from data including Iterative Dichotomiser 3 (ID3) [28], its successor—C4.5, Classification And Regression Tree (CART), Chi-square Automatic Interaction Detection (CHAID), and Multivariate Adaptive Regression Splines (MARS). This paper focus only on the CART implementation [29] available in Scikit-learn [30,31].

For CART, the use of the Gini index and a max depth of five were used as predefined parameters, as they provided a similar performance to the C4.5 algorithm. Contrary to C4.5, CART helped identify the most significant variables and to eliminate non-significant ones [32].

### 3.2. Multinomial Naïve Bayes

NB classifiers include several highly-scalable and simple probabilistic classifiers that rely on Bayes theorem with strict independence assumptions between features. When coupled with kernel density estimation they can achieve elevated classification accuracy levels [26].

The NB classifier is defined as:

(1) $$class_{nb}=\underset{class_{j}\in C}{\arg\;\max}\;p\left(class_{j}\right)\prod_{i}p\left(v_{i}|class_{j}\right),$$

where $p\left(v_{1},v_{2},\ldots ,a_{i},\ldots ,v_{17}|class_{j}\right)=\prod_{i}p\left(v_{i}|class_{j}\right)$, because this classifier assumes that the variables, $v_{i}$ are conditionally independent, given the class, and $class_{j}\in C$ are the classes or labels [33]. NB usage relied on calculations of the prior probabilities and estimation on the prior probabilities.

### 3.3. Multi Layer Perceptron (MLP)

**Figure 2:**
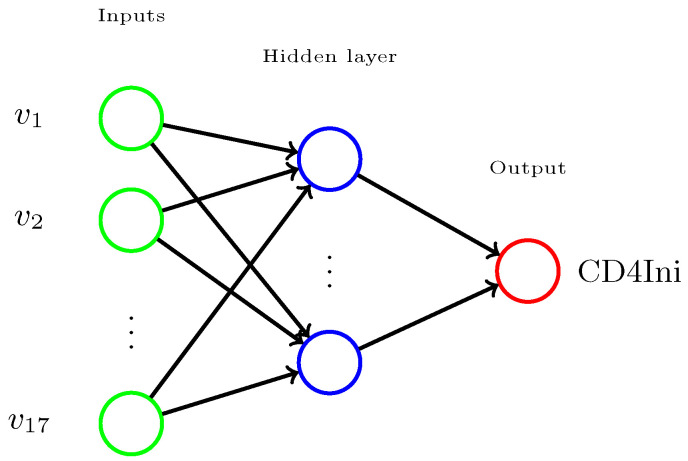
MLP architecture. There is a MLP per clinical endpoint; here is an example for CD4Ini.

MLP is based on classical ANN models, in particular the Perceptron introduced by F. Rosenblatt in 1957 [34]. MLP architecture is a more complex ANN where at least one or more hidden layers are included before the clinical endpoint variable layer [35]. MLP is also known as backpropagation [36,37,38,39], a generalization of the delta rule learning algorithm proposed by B. Widrow in 1962 [40]. MLPs are also referred to as feedforward neural networks. Figure 2 illustrates a general MLP architecture with $v_{1},v_{2},\ldots ,v_{17}$ input variables (green), a hidden layer (blue) and a single clinical endpoint (red). There is a single MLP for each of the clinical endpoint variable classes: CD4Ini, CD4Hist, VLIni, and VLHist.

For MLP training, we use the *logistic* activation function, a hidden layer with 8 neurons, 2 outputs, and 10,000 epochs with the Limited-memory BFGS algorithm (the Broyden–Fletcher–Goldfarb–Shanno algorithm), which is a method for numerical optimization [41].

### 3.4. Support Vector Machine (SVM)

SVMs are state-of-the-art algorithms initially introduced by Cortes and Vapnik as support-vector networks [42,43]. SVM were developed in an effort to develop artificial intelligence strategies for complex problems. SVM have mostly been applied to classification or regression problems. For classification purposes, SVMs aim to produce a mathematical *n*-dimensional space function capable of non-linearly distinguishing between different classes from complex and multivariate (training and test) datasets

Given a dataset

$$\begin{array}{c} D=\{\left(x^{1},y^{1}\right),\cdots ,\left(x^{l},y^{l}\right)\}, \end{array}$$

where $x\in R^{17}$ (inputs), y∈{−1,+1} (clinical endpoint), and *l* is the size of the dataset.

The SVM classifier is defined as

(2) $$f\left(x\right)=sgn\left(\sum_{i\in SVs}\alpha_{i}K\left(x_{i},x\right)\right)$$

which is a linear combination of kernels, $K(x_{i},x)$, where the sign function (sgn) gives the class [42]:

$$\begin{array}{c} \begin{array}{cccc} sgn: & R & \to & \left\{-1,0,1\right\}\\ & x & \to & y=sgn(x). \end{array} \end{array}$$

with constrains, $0\leq \alpha_{i}\leq C,i=1,\cdots ,l$, and $\sum_{j=1}^{l}\alpha_{j}y_{j}=0$. The parameter *C* is known as the margin and the Support Vectors (SV) will have non-zero Lagrange multipliers, $\alpha_{i}$; $K(x_{i},x_{j})$ is the kernel function performing the non-linear mapping into feature space ϕ, known as the “kernel trick” [26,42,43].

There are many kernel functions available for use with SVMs including linear, Gaussian Radial Basis Function (RBF), sigmoid, and polynomial. Our approach made use of the RBF kernel, where the width of a kernel is given by the γ parameter.

Across this research, SVMs used RBF as kernel with the following values: C=10 and γ=1.0.

### 3.5. Methods for Assessing the Relevance of Each Vif Variable

In order to assess the relevance that the different Vif variables (input) have on each of the included clinical endpoint variables (output), a series of steps were used, including:

1. Generating *p* balanced datasets through undersampling (see Section 2);
2. Constructing input variable combinations of less than 10 in size (*k*);
3. Identifying the variable combinations of each balanced datasets providing the best classification performance;
4. Calculating the relevance of each variable through a probabilistic approach, and;
5. Optimizing the selection of the most relevant variables by using a threshold value.

**Figure 3:**
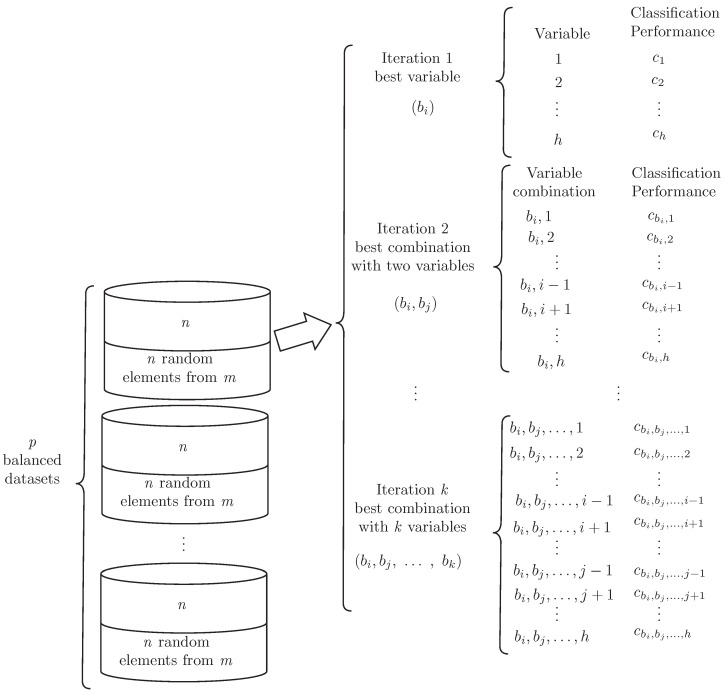
The process for defining the most relevant variables involves the search for the best combinations of variables including at most *k*-elements by using each balanced dataset. This search explores the interactions among the variables and their impact on the classification performance.

For the first step, balanced datasets are generated through undersampling by creating *p* partitions, which include all elements of the minority class (*n*) and an equal number of randomly selected elements of the majority class (i.e., *n* examples out of *m*), as shown in Figure 1. After producing balanced datasets, a second step addresses the construction of *k* size variable combinations by using each of them as input in different classification algorithms. For this, a five-fold cross-validation training process using weighted accuracy was used. The construction of the variable combinations relied on using greedy step-wise variable selection, as shown in Figure 3, in such a way as to identify the best variable capable of discriminating between the clinical endpoint classes. This process was repeated for a second variable in combination with the first identified and the process was repeated *k*-times so as to identify the *k* best variable combinations available.

**Figure 4:**
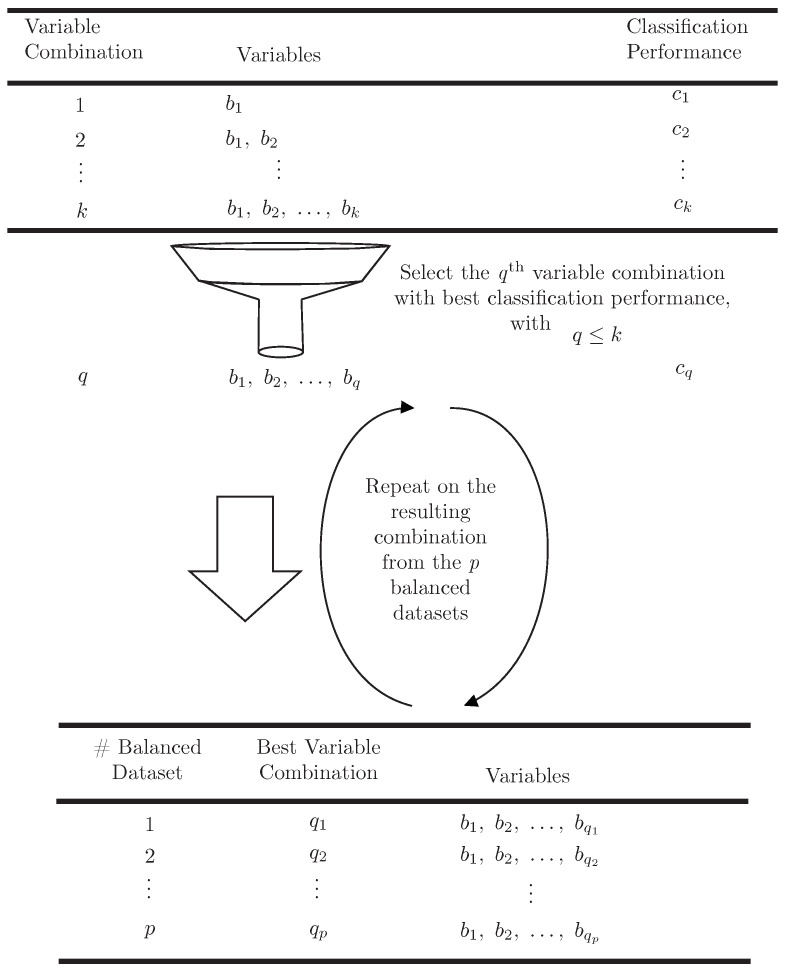
The selection of the overall-best combinations for each *p* balanced dataset by using their classification performance.

A third step involved discovering the best *k* combinations for each *p* balanced dataset. As the discovery of a global optimum is not guaranteed, a reasonably good local optimum (based on classification performance) was used, as shown in Figure 4. Global optimums are not realistically feasible as the search space exponentially explodes with *k*.

In a fourth step, variable relevance assessment is achieved using the *p* best combinations through a probabilistic approach. For this, the probability of each input Vif variable appearing at jth position on the variable combination matrix produced in the previous step is calculated using Equation (3).

(3) $$p\left(v_{i}^{j}\right)=\frac{f(v_{i}^{j})}{\sum_{a}f\left(v_{j}^{a}\right)}$$

where $p(v_{i}^{j})$ indicates the probability that the ith variable was selected at the jth position of the generated combinations. The frequencies for the variable and that of the different variables at the position jth are expressed as $f(v_{i}^{j})$. This equation is applied for each one of the *k* positions (j≤k). These probabilities define the relevance score (*r*) for each variable by using Equation (4):

(4) $$r_{i}=\sum_{j=1}^{k}\left(k+1-j\right)\times p\left(v_{i}^{j}\right)$$

where $r_{i}$ indicates the relevance score for the variable ith, considering its probability of appearing on each of the *k* positions in the combination matrix. This process assigns greater weight to the variables that are found closest to the root (lower entropy) of the combination matrix and less weight to those that appear farther from the root (higher entropy).

In a fifth step, the relevance scores obtained in the previous step are then used for sorting the variables considering their relevance scores and by establishing a threshold value (which involves calculating the upper limit of a 99% confidence interval of their relevance scores) to determine the most relevant variables (those surpassing the threshold limit).

#### 3.5.1. MAREV-1

The first Method for Assessing the Relevance of Each Variable (hereafter called MAREV-1) considers the classification results produced by each algorithm (CART, Multinomial NB, SVMs, and MLP) on p=100 balanced datasets. This yielded a total of 400 variable combinations having the highest classification performances, all of which were then tested further, including traditional statistical analysis, as mentioned below, see Section 3.5.3.

#### 3.5.2. MAREV-2

The second method, MAREV-2, selects only the best variable combinations assessed as classification performance for each algorithm (the third step described above), see Section 3.5. This yielded four input variable combinations, one per algorithm. Again, as mentioned above for the score assessment on each variable, all were then tested through the following traditional statistical analysis.

#### 3.5.3. Hypothesis Evaluation on the MAREV-1 and MAREV-2 Approaches

Once the most relevant variables had been identified in the previous steps, subsequent analysis involved establishing the clinical importance of the different machine learning algorithm-suggested variable combinations and their status (*Mut* or *Cons*) through traditional statistical association methods. For this, the Vif protein conserved sites, synonymous amino acid substitutions, or those being non-synonymous but conserved in physicochemical properties were encoded as “0” (*Cons* in the following discussion, figures, and tables). Contrarily, mutations leading to non-synonymous amino acid substitutions resulting in non-conserved physicochemical properties of the Vif protein (polar to non-polar changes, acidic to basic changes, gross molecular structure size changes, as well as changes in susceptibility to post-translational modifications such as phosphorilation, ubiquitination, SUMOylation, methylation, and glycosylation) were encoded as “1” (*Mut*). The definition of explicit variable-value combinations used the ID3 algorithm as implemented in the Waikato Environment for Knowledge Analysis (WEKA) workbench v3.6 [44]. ID3 was used for generating a decision tree for each clinical endpoint relying on tree branches to incorporate variable status (*Mut* or *Cons*) combinations. The calculation of the statistical significance of variable frequency differences between clinical endpoint groups relied on two-sided Fisher’s exact test using IBM SPSS Statistics (version 21, IBM Corporation, Armonk, NY, USA).

## 4. Results

The position of the Vif encoding region within the HIV-1 reference sequence HXB2, and the position and nomenclature of the Vif protein motifs and their putative ligands, is provided in Figure 1. The APOBEC-1 variable, corresponding to the N-terminal APOBEC3 binding site (${}^{14}$DRMR${}^{17}$), was excluded from the original dataset as it remained conserved.

### 4.1. Classification on the Balanced Datasets

The assessment of the relevance of each variable, as explained in Section 3.5, was based on the classification performance from four different classifiers (CART, MLP, SVMs, and Multinomial-NB) as implemented in the Scikit-learn package [30].

We have identified the top 100 variable-combinations associated to each clinical endpoint class by applying the proposed method to assess variable relevance. We obtained 1600 top-performing genetic variable-combinations associated to each clinical endpoint (CD4Ini, CD4Hist, VLIni, and VLHist) using the four classification algorithms. The balanced-accuracy was calculated with a 5-Cross-Validation approach during each training process. Algorithm accuracy was defined as the correct identification of both true positive and true negative registry examples (patients) and encompasses true-positive and true-negative predictive rates.

Out of the four machine learning algorithms tested, MLP superseded the three other machine learning algorithms during the analysis of each of the four clinical endpoints, accurately classifying, 79.6%, 76%, 68.5%, and 66.3% of CD4Ini, CD4HIts, VLIni, and VLHist patient registries, respectively. The classification performance of each machine learning algorithm for each clinical endpoint is summarized in Table 1.

Although the best classification results achieved higher values than those previously reported elsewhere [20], this can easily be explained by the use of balanced datasets and 5-Cross-Validation settings in this report. The genetic variable combinations providing the best classification performance are summarized in Table 2.

Considering the top scores per clinical endpoint shown in Table 2, the best discrimination was achieved for the CD4 T cells counts (CD4Ini and CD4Hist clinical endpoints).

On the other hand, low performance was observed on the VLIni clinical endpoint [71.5–80.2], and even lower for the VLHist [68.5–73.8].

Some variables were shown to be present in all “top combinations” identified for each different clinical endpoints. These were: [BCbox-3, BCbox-2, and APOBEC-2] for CD4Ini, [APOBEC-2, APOBEC-4, and BCbox-3] for CD4Hist, [APOBEC-2 and APOBEC-4] for VLIni, and [NLIS, APOBEC-2, and BCbox-1] for VLHist. Only the variable APOBEC-2 was present in 15 of the 16 best-combinations, except for in the combination with the highest classification when using MLP with the CD4Ini clinical endpoint. On the other hand, BCbox-3 was present in all the best combinations related to the CD4 T cell count.

### 4.2. Results Using the MAREV-1

After defining the 100 best-combinations per clinical endpoint by each algorithm, an assessment on the relevance of each variable was then undertaken. This involved calculating the probabilities for each variable of being selected as the most informative (i.e., root variable) in each of the best combinations. The relevance scores (*r*) per algorithm and positions are shown in Appendix A, see Table A1, Table A2, Table A3 and Table A4. After evaluating all the variables for each clinical endpoint, a threshold was calculated per clinical endpoint and used for selecting the most relevant variables as mentioned previously; see Section 3.5. The calculated threshold values for the most relevant variables are summarized in Appendix A, see Table A6a. The variables indicated as most relevant for CD4Ini (ordered by their relevance scores) were: [BCbox-3, APOBEC-3, APOBEC-5, APOBEC-2]; for CD4Hist: [APOBEC-2, APOBEC-3, APOBEC-5]; for VLIni they were [APOBEC-2, BCbox-1, APOBEC-3] and, finally; for VLHist they were [NLIS, APOBEC-3, APOBEC-5]. Considering these most relevant variables, APOBEC-3 proved to be associated with all the clinical endpoints, while APOBEC-2 and APOBEC-5 were present in only three clinical endpoints. BCbox-1 was seen to be the most relevant for only VLIni. BCbox-3 was only relevant for CD4Ini, and NLIS was suggested as being the most relevant in only VLHist.

The most relevant variables identified were in agreement with the best variables identified in previous efforts using alternative approaches [20], as shown in Table A7b; see Appendix A. This was also the case for the second variables in the clinical endpoints CD4Hist and VLIni. Another difference was that the quantity of variables defined as the most relevant when using the MAREV-1 approach was much higher for the clinical endpoints CD4Ini and CD4Hist than reported previously.

### 4.3. Results Using the MAREV-2

In this approach, the variable assessment process was done considering only the combinations of variables having the best classification performance, see Table 2. As happens with MAREV-1, MAREV-2 also calculated the probability for each variable to appear at every available position. This was later used to determine the score per variable and clinical endpoint as shown in Table A6b); see Appendix A. The variables discovered to be more relevant for CD4Ini (ordered by their scores) were: [BCbox-3, BCbox-2]; [APOBEC-2, APOBEC-4, BCbox-3] for CD4Hist; [APOBEC-2, APOBEC-4, BCbox-1] for VLIni; and [APOBEC-2, NLIS, BCbox-1] for VLHist. None of the variables were shown to be present in all clinical endpoints unlike MAREV-1. However, APOBEC-2 was present in CD4Hist, VLIni and VLHist. On the other hand, APOBEC-2 and APOBEC-4 are related to CD4Hist and VLIni; BCbox-1 is relevant for VLIni and VLHist. Finally, BCbox-3 is relevant for CD4Ini and CD4Hist. BCbox-2 is only relevant for CD4Ini, while NLIS is relevant for VLHist. These variables are compared with the previous findings and those suggested by the 100-model analysis (see Table A7c in Appendix A).

The comparison among the variables identified as the most relevant by the previous approach, MAREV-1 and MAREV-1, show a coincidence in some of the variables detected as most relevant. This is the case of BCBox-3 in CD4Ini and APOBEC-2 in both CD4Hist and VLIni. Although MAREV-1 and the previous approach agreed on assigning NLIS as the most relevant variable for VLHist, this motif was only suggested as the second most relevant for this clinical endpoint by MAREV-2.

### 4.4. Decision Trees and the Most Relevant Variable Combinations from MAREV-1 and MAREV-2

**Figure 5:**
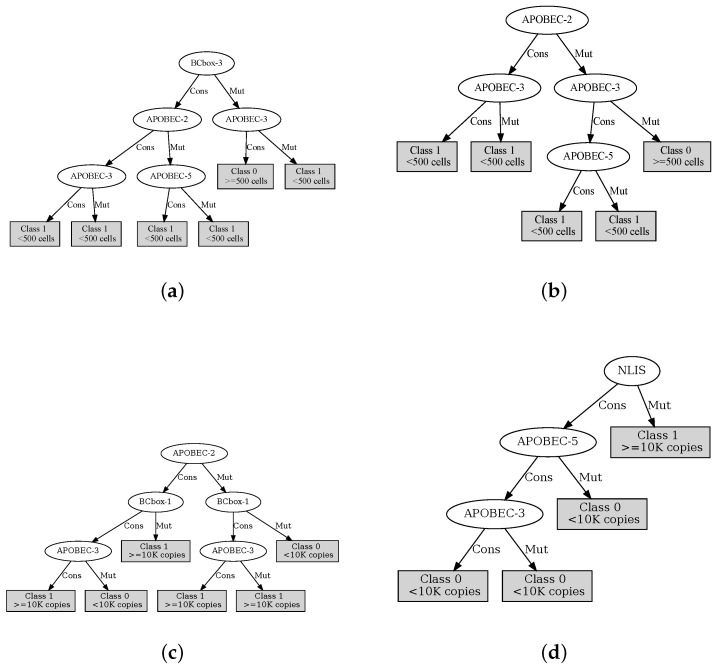
ID3 inducted trees using the selected most relevant variables per output as defined by the MAREV-1 approach. (**a**) The tree for CD4Ini; (**b**) The tree for CD4Hist; (**c**) The tree for VLIni; (**d**) The tree for VLHist.

**Figure 6:**
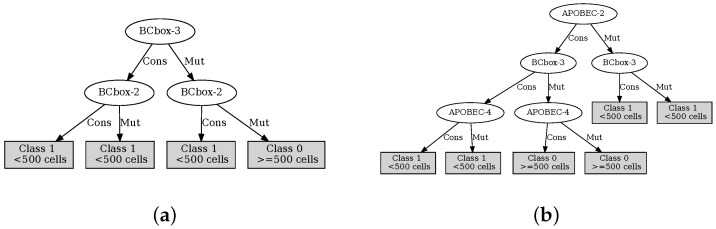

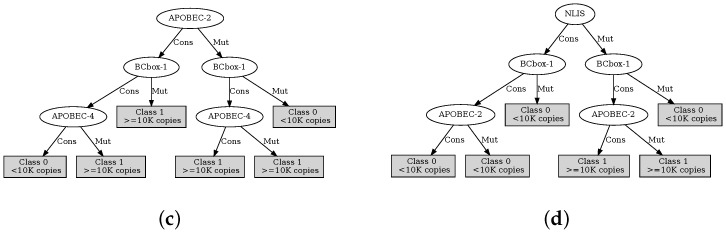
ID3 inducted trees using the suggested most relevant variables per output as defined by the MAREV-2 approach. (**a**) The tree for CD4Ini; (**b**) The tree for CD4Hist; (**c**) The tree for VLIni; (**d**) The tree for VLHist.

The decision trees defined with the variables determined by the MAREV-1 are shown in Figure 5, while those using the MAREV-2 are shown in Figure 6.

ID3 branch frequency was used to identify specific combinations of input variable status (${}^{Mut}$ or ${}^{Cons}$) as related to the clinical endpoints in Fisher’s exact test. Only branches having more than 1 variable were considered, yielding a total of 20 variable combinations for the MAREV-1 approach (6 for CD4Ini, 5 for CD4Hist, 6 for VLIni, and 3 for VLHist) whereas the MAREV-2 approach identified 22 different relevant variable combinations (4 for CD4Ini, 6 for CD4Hist, 6 for VLIni, and 6 for VLHist. The results of the statistical assessment for the MAREV-1 and MAREV-2 approaches are shown in Table 3.

Four of the 20 ID3-combinations defined from the MAREV-1 approach were detected as associated with clinical endpoints after further statistical testing. One was present for CD4Ini (*p*-value =0.0011), two for CD4Hist (*p*-value =0.0136, *p*-value =0.0182), and one for VLIni (*p*-value =0.0207). None of the associated combinations were present in VLHist. The combination for CD4Ini [BCboc-3${}^{Mut}$, APOBEC-3${}^{Cons}$] suggests protection from having lower numbers of CD4 T lymphocytes at the time of initial medical assessment as it was present in only 6 patient samples having ≤500 CD4 T cells, compared to 53 patient samples not having said combination. In the case of CD4Hist, only one combination [APOBEC-2${}^{Cons}$, APOBEC-3${}^{Cons}$] suggested protection from having less than 500 T Lymphocytes on medical follow-up, as was also found in our previously published work. A second combination [APOBEC-2${}^{Mut}$, APOBEC-3${}^{Cons}$, APOBEC-5${}^{Cons}$] was found to be associated with the risk of progression to less than 500 CD4 T lymphocytes on medical follow-up. The absence of said combination was detected in 14 out of 15 sequences with ≥500 CD4 T cells. Finally, in the case of VLIni, the [APOBEC-2${}^{Mut}$, BCbox-1${}^{Cons}$, APOBEC-3${}^{Cons}$] combination suggested a risk of having higher HIV viral loads on the first medical examination as it was absent in 22 out of the 26 cases with less than 10,000 virus copies.

On the other hand, the 22 ID3-combinations generated using the variables defined by the MAREV-2 yielded 5 clinical associations. Both of the associations found in CD4Ini involved variables BCBox-2 and BCBox-3 where the conservation of both protein regions was associated with a higher risk of having lower initial CD4 T lymphocytes on the first medical examination (*p*-value =0.0068). This variable combination was present in 26 of the patient cases with <500 CD4 cells/μL, compared with a single occurrence in a patient having ≥500. A second variable combination, [BCBox-2${}^{Mut}$ and BCBox-3${}^{Mut}$], was associated with protection from low CD4 T lymphocytes counts as it was observed to be more frequent in patients having ≥500 CD4 cell count/μL (*p*-value =0.0049). Regarding historic CD4 T cell counts, one variable combination [APOBEC-2${}^{Mut}$, BCbox-3${}^{Cons}$] was associated with the risk of having low CD4 T cell counts on medical follow-up as it was present in 20 cases with a CD4 cell count below 500 and not in patients having ≥500 CD4 T cells/μL. Regarding initial viral load assessments, [APOBEC-2${}^{Mut}$, APOBEC-4${}^{Mut}$, BCbox-1${}^{Cons}$] was associated with the risk of having high viral titres (≥10,000 viral copies) at the time of initial medical examination and was present in 11 patients having ≥10,000 viral copies, yet in only a single patient having lower viral loads. Finally, [NLIS${}^{Mut}$, APOBEC-2${}^{Mut}$, BCbox-1${}^{Cons}$] was observed to be associated with a higher risk of low historical viral loads on patient follow-up as it was seen only once in a patient having <10,000 copies but it was present in 6 patients having more than 10,000 copies of the virus. As mentioned before, eight novel HIV associations were identified through this approach: three by MAREV-1, and five with MAREV-2.

Distinct Vif protein regions were identified through this approach as being highly relevant by MAREV-1, mainly involved in APOBEC3 interactions and Elongin B/C binding. Relevant APOBEC3 interaction motifs included APOBEC-3, which was found to be conserved in all cases as well as APOBEC-2, which only failed to be relevant with regard to CD4Ini. Similarly, APOBEC-5 was found to be absent in CD4Hist while BCbox-1 was related to VLIni. Similarly, MAREV-2 also identified APOBEC-3, APOBEC-2, and APOBEC-4, and the Elongin B/C-box binding motifs, BCbox-1, BCbox-2, and BCbox-3 as most relevant. The results from the MAREV-2 for VLHist agree with our previously published findings by suggesting a higher relevance of the NLIS segment.

These results help supporting the variables detected as more informative in our previous findings [20], being: (i) [BCbox-3] for CD4Ini, (ii) [APOBEC-2] for CD4Hist, VLIni and VLHist, (iii) [BCbox-1] for VLIni and VLHist, and iv) [NLIS] for VLHist. Additionally, the MAREV-1 approach places relevance for the variables [APOBEC-3 and APOBEC-5] while MAREV-2 places relevance for [APOBEC-4, BCbox-2, and BCbox-3]. On the other hand, the four associations determined with MAREV-1 and the five determined by MAREV-2 were less than the seven suggested with the previously methodology. Only one of said associations was present when using both approaches. Fewer associations were found when considering the viral load clinical status, both the initial and historical. This was the case for VLHist, where no association was found when using the MAREV-1 approach. However, determining which set of associations have more biological significance requires further research.

Table 3 concentrates the most relevant associations of genetic variable combinations with each of the four clinical endpoint variables out of the 20 and 22 hypotheses tested by the MAREV-1 and MAREV-2 algorithms, respectively. On initial examination, the reiterative appearance of APOBEC and Elongin B/C Box motifs stands out in the results generated by both algorithms, irrespective of site status (mutated or conserved). This is a reflection of the importance of Vif protein, a function which involves both binding of Elongin B/C and recognition of APOBEC molecules to provide HIV with the capacity to escape from APOBEC-mediated innate immunity. From within the eight different APOBEC binding sites included in the analysis, APOBEC-2 and APOBEC-3 stand out for the number of times they appear in the associations shown in Table A5. Interestingly, the APOBEC-2 and -3 sites bind APOBEC3G and APOBEC3F, the two most relevant members of the APOBEC3 family of antiviral proteins. Nevertheless, our results are indicative that the APOBEC3G and APOBEC3F protein binding site (APOBEC-2) is perhaps the least important of all the genetic Vif variables assessed. This is based on the fact that both MAREV-1 and MAREV-2 results show higher viral titres and lower CD4 T cell numbers (suggesting ongoing viral robustness) even in the presence of APOBEC-2 mutations, as long as the other APOBEC-binding regions or Elongin B/C binding regions remain conserved. This was observed in historic CD4 T cell numbers, the initial viral loads, and regarding the historic viral loads.

**Figure 7:**
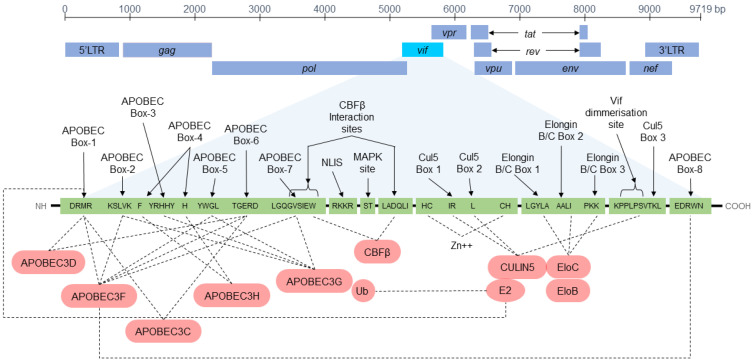
Position of the Vif encoding region within a reference (HXB2) HIV-1 genome.

Similarly, the recursive appearance of Elongin B/C box-1 and box-3 binding sites also highlights the relevance that the Elongin interactions have for the Vif protein mediated ubiquitination of APOBEC3 anti-viral proteins. Overall, our results emphasize the clinical relevance of both APOBEC3G and Elongin B/C binding sites from among the remaining Vif protein domains assessed. Figure 7 illustrates the position of the Vif encoding region within a reference (HXB2) HIV-1 genome, the Vif protein domains and regions, as well as some of the putative or known ligands. Even greater detail is provided by our results regarding the weight of each of these genetic variables when individual clinical outcomes are considered. At least one previous report has identified that amino acid substitutions in Elongin B/C sites lead to a loss-of-infectivity in HIV [45].

The results of both MAREV-1 and -2 suggest that initial CD4 T cell numbers seem to depend more on Elongin B/C site status than any other Vif protein attribute. When Elongin B/C box mutations are present, such as in [BCbox-3${}^{Mut}$, APOBEC-3${}^{Cons}$] (MAREV-1) and [BCbox-3${}^{Mut}$, BCbox-2${}^{Mut}$] (MAREV-2), a greater number of patients are seen to be present in the ≥500 cells/μL class than in the ≤500 cells/μL class. This supports the notion that Elongin B/C binding box mutations are detrimental to viral fitness and thus prevent HIV from escaping APOBEC3 inhibition or interference.

An additional interesting finding relates to historic CD4 T cell numbers and viral loads. HIV patients are normally enrolled into anti-retroviral therapy protocols after being diagnosed, irrespective of CD4 T cell counts and viral load numbers. The clinical impact that viral mutations have at this stage, after initiating treatment, has largely been linked to protease, reverse-transcriptase, and integrase sites, those most subjected to selective pressures by anti-retroviral drugs. Our results indicate that the conservation of APOBEC binding motifs are essential to viral fitness (and worsening clinical progression), at least in the MAREV-1 results. As such, [APOBEC-2${}^{Mut}$, APOBEC-3${}^{Cons}$, APOBEC-5${}^{Cons}$] and [APOBEC-2${}^{Cons}$, APOBEC-3${}^{Cons}$] were more common among patients having lower CD4 T Cell numbers on follow-up. This was also true for BCbox-3 in MAREV-2 results, where [APOBEC-2${}^{Mut}$, BCbox-3${}^{Cons}$] was also more common among patients having ≤500 cells/μL. Previous reports have highlighted how the conservation of APOBEC binding sites is crucial for vif-mediated viral fitness. Our results suggest that the mutation of certain APOBEC3 binding site motifs (i.e., APOBEC-2) is tolerated without a significant effect on viral fitness as long as other, perhaps more important, remaining motifs are conserved (i.e., APOBEC-3 and or -5) [46].

## 5. Conclusions

This paper proposes a new methodology based on machine learning algorithms (CART, NB, SVMs, and MLP) combined with an undersampling approach to deal with an imbalanced HIV dataset. Additionally, we present evidence of the classification performance of two different approaches (MAREV-1 and MAREV-2) for the identification of associations of Vif protein motifs with clinical endpoints in HIV. These variables subsequently proved to play a crucial role when different combinations of them were linked to HIV outcome, a difficult task that is not possible to achieve in human terms without relying on statistical corrections that decrease the statistical power of the study. These findings are in agreement with the known properties and with the functional and clinical relevance of the different Vif protein motifs found to be relevant. Needless to say, further research employing cell biology and molecular epidemiology tools is warranted so as to provide further support for these claims. Efforts are currently underway in our group to test the clinical utility of the identified variable combinations in a novel, larger HIV cohort.

When comparing the different strategies described in this manuscript, MAREV-2 was able to identify many more clinical associations, at least one per clinical outcome. This might be interpreted to suggest that this approach might prove more useful in future analysis and in clinical settings.

Many techniques are currently available to deal with imbalanced datasets. Although we studied the capacity of an undersampling approach to resolve this limitation, future work will explore the performance of oversampling techniques. These results provide further evidence on the usefulness and potential that machine learning methods have at analyzing complex datasets. Given the exponential growth of applications of artificial intelligence and classification strategies, this field is likely to benefit from the results presented herein.

Elongin B/C binding site mutations might prove to be the single most important Vif genetic feature determining CD4 T cell numbers at the time of clinical debut and at a time when viral replication has not been subjected to the influence of anti-retroviral drugs (as patients are treatment-naïve at this time). This opens the possibility that molecular approaches targeting HIV-1 Elongin B/C binding motifs or those inhibiting the interactions of Elongin B/C and Vif might provide innovative preventative strategies in the fight against HIV.

Overall, our results provide insight into the utility that both MAREV-1 and -2 algorithms have at discriminating complex genetic variable combinations linked to clinical endpoints in HIV, the practical utility of screening for accessory protein encoding region mutations in HIV prognosis, as well as at guiding the development of novel therapeutic interventions in HIV.

## Figures and Tables

**Table 1:** Summary of the performance of the algorithm considering 100 runs on the balanced datasets for each clinical endpoint in descending order of their mean value. Our results demonstrate that MLP produced the best classification performance for all the comparisons made.

Clinical Endpoint	Algorithm	Mean/S.D.	Range		Clinical Endpoint	Algorithm	Mean/S.D.	Range
CD4Ini	**MLP**	**79.6 ± 5.7**	**68.6–93.8**		VLIni	**MLP**	**68.5 ± 3.2**	**61.1–75.2**
	CART	77.8 ± 6.0	65.7–91.0			CART	68.0 ± 3.7	59.1–80.2
	SVMs	76.2 ± 5.6	61.0–88.1			NB	66.5 ± 3.4	57.4–75.0
	NB	74.9 ± 5.9	59.5–90.5			SVMs	62.0 ± 4.0	51.7–71.5
CD4Hist	**MLP**	**76.0 ± 5.4**	**63.3–91.0**		VLHist	**MLP**	**66.3 ± 2.7**	**60.9–73.8**
	CART	74.0 ± 6.2	62.9–88.1			CART	64.2 ± 2.5	59.1–71.4
	NB	72.6 ± 5.8	60.0–87.6			NB	64.1 ± 2.9	57.5–71.1
	SVMs	66.7 ± 6.4	53.8–81.9			SVMs	63.2 ± 3.0	51.3–68.5

**Table 2:** Best classification performance achieved by each algorithm, considering 100 balanced datasets for each clinical endpoint. These combinations were used for calculating the variables scores with the MAREV-2 approach.

Clinical Endpoint	Algorithm	Combination	Accuracy
CD4Ini	MLP	BCbox-3, APOBEC-3, BCbox-2, Cul5-3, BCbox-1, APOBEC-5	93.8
	CART	BCbox-3, BCbox-2, Cul5-3, APOBEC-2, APOBEC-3, APOBEC-5	91.0
	NB	APOBEC-2, BCbox-3, APOBEC-3, BCbox-2, Cul5-3, APOBEC-6	90.5
	SVMs	BCbox-2, APOBEC-2, APOBEC-3, APOBEC-4, BCbox-1, BCbox-3	88.1
CD4Hist	MLP	APOBEC-2, Cul5-3, APOBEC-4, BCbox-3, APOBEC-7, BCbox-2, NLIS, BCbox-1	91.0
	CART	APOBEC-2, BCbox-3, BCbox-2, APOBEC-4, APOBEC-5	88.1
	NB	APOBEC-2, APOBEC-4, Cul5-3, CBFb-2, BCbox-3, APOBEC-7	87.6
	SVMs	APOBEC-2, APOBEC-3, APOBEC-4, APOBEC-5, APOBEC-6, APOBEC-8, APOBEC-7, BCbox-3	81.9
VLIni	CART	APOBEC-2, BCbox-1, APOBEC-4, BCbox-2	80.2
	MLP	APOBEC-2, BCbox-1, APOBEC-8, APOBEC-3, APOBEC-4, APOBEC-5, Cul5-2	75.2
	NB	APOBEC-2, APOBEC-4, BCbox-1, BCbox-2, NLIS, Cul5-3, APOBEC-3, APOBEC-5, CBFb-1	75.0
	SVMs	APOBEC-2, APOBEC-7, APOBEC-3, APOBEC-4, APOBEC-5, APOBEC-6, APOBEC-8, BCbox-2	71.5
VLHist	MLP	NLIS, APOBEC-3, APOBEC-2, APOBEC-8, BCbox-1, CBFb-1, Cul5-1, Cul5-2	73.8
	CART	APOBEC-2, BCbox-3, BCbox-1, APOBEC-8, NLIS total	71.4
	NB	APOBEC-2, Cul5-3, NLIS, BCbox-2, APOBEC-3, BCbox-1, APOBEC-8, CBFb-2, APOBEC-6, APOBEC-7	71.1
	SVMs	NLIS, APOBEC-4, BCbox-1, APOBEC-2, APOBEC-5, APOBEC-6, BCbox-3	68.5

**Table 3:** The most relevant Vif protein variable combinations associated with the clinical endpoints. (a) Significant associations after testing the 20 hypothesis suggested by the MAREV-1 approach; (b) Significant associations after testing the 22 hypothesis suggested by the MAREV-2 approach. Vif protein regions can either be conserved (Cons) or mutated (Mut) and associated with protection (*prot*) or *risk* to either <500 cells/μL CD4 T cells or ≥10,000 cp/mL of viral load.

				Contingency Tables		Classification		
Approach	Output	Vif Variable Combination	Status	≥500 cells/μL	<500 cells/μL	Accuracy	Error	*p*-Value^*effect*^
*(a)* MAREV-1	Initial CD4	BCbox-3${}^{\mathrm{Mut}}$, APOBEC-3${}^{\mathrm{Cons}}$	absent	8	53	81.3%	18.7%	0.0011${}^{\mathrm{prot}}$
			present	8	6	(61/75)	(14/75)	
	Historic CD4	APOBEC-2${}^{\mathrm{Mut}}$, APOBEC-3${}^{\mathrm{Cons}}$, APOBEC-5${}^{\mathrm{Cons}}$	absent	14	35	52.0%	48.0%	0.0136${}^{\mathrm{risk}}$
			present	1	25	(39/75)	(36/75)	
		APOBEC-2${}^{\mathrm{Cons}}$, APOBEC-3${}^{\mathrm{Cons}}$	absent	2	29	56%	44.0%	0.0182${}^{\mathrm{prot}}$
			present	13	31	(42/75)	(33/75)	
				<10,000 cp/mL	≥10,000 cp/mL			
	Initial VL	APOBEC-2${}^{\mathrm{Mut}}$, BCbox-1${}^{\mathrm{Cons}}$, APOBEC-3${}^{\mathrm{Cons}}$	absent	22	28	57.3%	42.7%	0.0207${}^{\mathrm{risk}}$
			present	4	21	(43/75)	(32/75)	
	Historic VL	—– —– —– —– —– —– —– —–	—	—	—	—	—	—
*(b)* MAREV-2	Initial CD4	BCbox-3${}^{\mathrm{Cons}}$, BCbox-2${}^{\mathrm{Cons}}$	absent	15	33	54.7%	45.3%	0.0068${}^{\mathrm{risk}}$
			present	1	26	(41/75)	(34/75)	
		BCbox-3${}^{\mathrm{Mut}}$, BCbox-2${}^{\mathrm{Mut}}$	absent	10	55	81.3%	18.7%	0.0049${}^{\mathrm{prot}}$
			present	6	4	(61/75)	(14/75)	
	Historic CD4	APOBEC-2${}^{\mathrm{Mut}}$, BCbox-3${}^{\mathrm{Cons}}$	absent	15	40	53.3%	46.7%	0.0077${}^{\mathrm{risk}}$
			present	0	20	(40/75)	(35/75)	
				<10,000 cp/mL	≥10,000 cp/mL			
	Initial VL	APOBEC-2${}^{\mathrm{Mut}}$, BCbox-1${}^{\mathrm{Cons}}$, APOBEC-4${}^{\mathrm{Mut}}$	absent	25	38	52.0%	48.0%	0.0477${}^{\mathrm{risk}}$
			present	1	11	(39/75)	(36/75)	
	Historic VL	NLIS${}^{\mathrm{Mut}}$, BCbox-1${}^{\mathrm{Cons}}$, APOBEC-2${}^{\mathrm{Mut}}$	absent	41	27	62.7%	37.3%	0.0392${}^{\mathrm{risk}}$
			present	1	6	(47/75)	(28/75)	

## Data Availability

The dataset used in this research was already published [23], and it is publicly available at http://www.genomica.uaslp.mx/Research/HIV.html, accessed on 28 October 2022.

## Abbreviations

The following abbreviations are used in this manuscript:

HIV	Human Immunodeficiency Virus
Vif	Viral Infectivity Factor
CD4	Cluster of Differentiation 4
APOBEC3	APOlipoprotein Bmessenger RNA Editing enzyme, Catalytic polypeptide-like
NLIS	Nuclear Localisation Inhibitory Signal
SVMs	Support Vector Machines
ANNs	Artificial Neural Networks
NB	Naïve Bayes
MLP	Multi-Layer Perceptron
RBF	Radial Basis Function

## Appendix A. Variable Assessment

Following the proposed methodology for assessing the relevance of each variable (see Section 3.5), the results from the fourth step are in Table A1, Table A2, Table A3 and Table A4. There is a table per clinical endpoint CD4Ini, CD4Hist, VLIni, and VLHist, respectively. Each table shows the results from each classification algorithm: CART, MLP, NB, and SVMs.

**Table A1:** Relevance scores (*r*) in a descending order per algorithm and variable considering the clinical endpoint **CD4Ini** using the MAREV-1 approach. The four variables with higher values are highlighted in bold.

(a) CART												
Rank	Variable	pos1	pos2	pos3	pos4	pos5	pos6	pos7	pos8	pos9	pos10	Total
**1**	**BCbox-3**	**6**	**1.355**	**0.545**	**0**	**0.188**	**0**	**0**	**0.107**	**0.087**	**0**	**8.282**
**2**	**APOBEC-3**	**0**	**2.71**	**1.818**	**1.793**	**0.469**	**0.192**	**0**	**0**	**0**	**0**	**6.982**
**3**	**APOBEC-5**	**0**	**0**	**2**	**1.11**	**1.875**	**0.673**	**0.186**	**0.107**	**0.087**	**0**	**6.038**
**4**	**APOBEC-2**	**0**	**2.613**	**1.364**	**0.768**	**0.562**	**0.288**	**0.186**	**0**	**0**	**0**	**5.782**
5	BCbox-2	2.4	1.161	0.273	0.939	0.094	0.096	0.093	0	0.348	0.1	5.504
6	APOBEC-6	0	0	0	0.939	0.469	1.346	0.744	0.321	0.435	0	4.254
7	APOBEC-4	1.6	0.097	0.455	0.427	0.656	0.096	0.093	0	0.087	0	3.511
8	Cul5-3	0	0	1.091	0.427	0.562	0.385	0.279	0	0.087	0.2	3.031
9	CBFb-1	0	0	0	0.085	0.281	0.673	0.465	0.964	0.348	0.1	2.917
10	APOBEC-7	0	0.097	0.091	0.341	0.375	0.288	0.837	0	0	0.3	2.33
11	APOBEC-8	0	0	0.091	0.171	0.094	0.288	0.279	0.536	0.174	0	1.633
12	NLIS	0	0.968	0.182	0	0	0.192	0	0.107	0	0	1.449
13	CBFb-2	0	0	0.091	0	0.188	0.096	0.279	0.214	0	0.1	0.968
14	Cul5-1	0	0	0	0	0.094	0.096	0.372	0.214	0.087	0.1	0.963
15	Cul5-2	0	0	0	0	0.094	0.096	0.093	0.321	0.087	0.1	0.791
16	BCbox-1	0	0	0	0	0	0.192	0.093	0.107	0.174	0	0.566
(**b**) **MLP**												
**Rank**	**Variable**	**pos1**	**pos2**	**pos3**	**pos4**	**pos5**	**pos6**	**pos7**	**pos8**	**pos9**	**pos10**	**Total**
**1**	**BCbox-3**	**7.3**	**0.09**	**0.33**	**0.151**	**0**	**0.07**	**0.07**	**0**	**0**	**0.125**	**8.136**
**2**	**APOBEC-3**	**0**	**2.79**	**2.062**	**0.527**	**0.714**	**0.211**	**0.14**	**0.071**	**0**	**0**	**6.516**
**3**	**APOBEC-5**	**0**	**0**	**1.732**	**1.355**	**0.643**	**0.915**	**0.211**	**0.071**	**0.069**	**0.062**	**5.059**
**4**	**BCbox-2**	**1.2**	**1.44**	**0.495**	**0.527**	**0.357**	**0.211**	**0.211**	**0**	**0.069**	**0.062**	**4.572**
5	APOBEC-2	0	2.07	0.825	0.527	0.357	0.282	0.14	0.143	0	0.062	4.406
6	APOBEC-4	1.3	0.09	0.577	0.903	0.286	0.282	0.211	0	0.069	0.062	3.78
7	Cul5-2	0	0	0.082	0.376	1.714	0.352	0.281	0.286	0.138	0.062	3.292
8	APOBEC-7	0.1	1.35	0.412	0.301	0.143	0.423	0.351	0.071	0.069	0.062	3.283
9	Cul5-3	0	0.09	0.66	0.602	0.357	0.282	0.421	0.357	0.345	0	3.114
10	APOBEC-6	0	0.09	0.33	0.828	0.357	0.563	0.281	0.286	0.138	0.188	3.06
11	APOBEC-8	0	0.09	0	0.226	0.5	0.352	0.421	0.143	0.207	0	1.939
12	CBFb-1	0	0	0	0.075	0.214	0.423	0.632	0.357	0	0.125	1.826
13	NLIS	0.1	0.72	0.165	0.151	0.071	0.211	0.14	0.071	0.069	0.062	1.761
14	CBFb-2	0	0.09	0.165	0.301	0	0.141	0.14	0.357	0.276	0.062	1.533
15	BCbox-1	0	0.09	0.165	0.075	0.214	0.211	0.211	0.286	0.138	0	1.39
16	Cul5-1	0	0	0	0.075	0.071	0.07	0.14	0.5	0.414	0.062	1.334
**(c) NB**												
**Rank**	**Variable**	**pos1**	**pos2**	**pos3**	**pos4**	**pos5**	**pos6**	**pos7**	**pos8**	**pos9**	**pos10**	**Total**
**1**	**APOBEC-2**	**10**	**0**	**0**	**0**	**0**	**0**	**0**	**0**	**0**	**0**	**10**
**2**	**BCbox-3**	**0**	**6.48**	**0.08**	**0.298**	**0.468**	**0.317**	**0.186**	**0.077**	**0**	**0**	**7.906**
**3**	**APOBEC-3**	**0**	**0.27**	**1.92**	**0.968**	**1.091**	**0.952**	**0.372**	**0**	**0**	**0.1**	**5.673**
**4**	**APOBEC-4**	**0**	**0.09**	**2.08**	**0.968**	**0.545**	**0.397**	**0.186**	**0.308**	**0.143**	**0**	**4.717**
5	BCbox-2	0	0.09	1.12	1.117	0.156	0.317	0.279	0.308	0.143	0	3.53
6	APOBEC-6	0	0	0.64	0.521	0.779	0.556	0.186	0.308	0.286	0	3.276
7	CBFb-2	0	0.63	0.72	0.298	0.312	0.159	0.186	0.231	0.143	0.1	2.778
8	APOBEC-5	0	0	0.08	0.223	0.701	0.476	0.651	0.231	0.286	0.1	2.749
9	APOBEC-7	0	1.08	0.08	0.447	0.312	0.238	0.093	0.308	0.143	0	2.7
10	Cul5-3	0	0	0.64	0.521	0.156	0.238	0.744	0.154	0.143	0	2.596
11	NLIS	0	0.27	0.08	0.521	0.234	0.397	0.279	0.154	0.143	0.2	2.278
12	BCbox-1	0	0	0.4	0.223	0.623	0.397	0.093	0.231	0.143	0.1	2.21
13	CBFb-1	0	0	0	0.223	0.39	0.238	0.279	0.385	0	0.2	1.715
14	APOBEC-8	0	0.09	0.16	0.372	0.156	0.159	0.279	0.154	0.143	0	1.513
15	Cul5-2	0	0	0	0.223	0.078	0	0.186	0.077	0	0.2	0.764
16	Cul5-1	0	0	0	0.074	0	0.159	0	0.077	0.286	0	0.596
(**d**) **SVMs**												
**Rank**	**Variable**	**pos1**	**pos2**	**pos3**	**pos4**	**pos5**	**pos6**	**pos7**	**pos8**	**pos9**	**pos10**	**Total**
**1**	**BCbox-3**	**5.5**	**0.827**	**0.774**	**0.402**	**0.676**	**0.323**	**0**	**0.083**	**0**	**0**	**8.585**
**2**	**BCbox-2**	**1.5**	**2.02**	**1.29**	**0.805**	**0.423**	**0.081**	**0.226**	**0**	**0.286**	**0**	**6.631**
**3**	**APOBEC-4**	**2.6**	**0.276**	**1.118**	**0.885**	**0.761**	**0.323**	**0.226**	**0.167**	**0**	**0.1**	**6.455**
**4**	**APOBEC-2**	**0.1**	**1.745**	**1.376**	**0.563**	**0.423**	**0.726**	**0**	**0.417**	**0.19**	**0.1**	**5.64**
5	APOBEC-3	0	0.276	1.032	2.011	0.676	1.048	0.302	0.167	0.095	0	5.607
6	APOBEC-7	0	2.663	0.688	0.483	0.507	0.242	0.226	0.083	0.19	0	5.083
7	NLIS	0.1	0.643	1.032	0.483	0.169	0.242	0.528	0.083	0.19	0	3.471
8	APOBEC-5	0	0.092	0.086	0.241	0.592	0.645	0.906	0.583	0.19	0	3.335
9	Cul5-3	0	0.092	0.258	0.483	0.676	0.565	0.528	0.083	0.19	0	2.875
10	APOBEC-6	0	0	0	0.161	0.423	0.242	0.679	0.583	0.19	0.2	2.478
11	CBFb-2	0.1	0.367	0.258	0.322	0	0.242	0.075	0	0.19	0	1.555
12	APOBEC-8	0.1	0	0.086	0.08	0.338	0.161	0.075	0.25	0.095	0.2	1.387
13	BCbox-1	0	0	0	0.08	0.169	0.081	0.075	0.167	0	0.1	0.672
14	Cul5-2	0	0	0	0	0.169	0.081	0.075	0.167	0.095	0	0.587
15	CBFb-1	0	0	0	0	0	0	0	0.167	0.095	0.3	0.562
16	Cul5-1	0	0	0	0	0	0	0.075	0	0	0	0.075

**Table A2:** Relevance scores (*r*) in descending order per algorithm and variable considering the clinical endpoint **CD4Hist** using the MAREV-1 approach. The three variables with higher values are highlighted in bold.

(a) CART												
Rank	Variable	pos1	pos2	pos3	pos4	pos5	pos6	pos7	pos8	pos9	pos10	Total
**1**	**APOBEC-2**	**6.8**	**0.2**	**0.494**	**0.467**	**0.338**	**0.094**	**0**	**0**	**0**	**0**	**8.393**
**2**	**APOBEC-3**	**0**	**4.8**	**0.691**	**0.467**	**0.676**	**0**	**0.133**	**0**	**0**	**0**	**6.767**
**3**	**APOBEC-5**	**0**	**0.4**	**1.481**	**2.24**	**0.845**	**0.377**	**0.4**	**0.214**	**0**	**0**	**5.958**
4	BCbox-3	2.1	1.3	0.889	0.653	0.169	0.189	0	0	0.167	0	5.467
5	APOBEC-4	0	0.4	2.765	0.28	0.761	0.472	0.133	0.214	0.333	0	5.359
6	Cul5-3	0.5	0.8	0.198	0.653	0.254	0.66	0.4	0.536	0.167	0	4.167
7	APOBEC-6	0	0.3	0.099	1.12	1.183	0.472	0.4	0.321	0	0.111	4.006
8	APOBEC-7	0.1	0	0.395	0.093	0.93	0.377	0.533	0.214	0	0.222	2.865
9	CBFb-1	0	0	0	0.093	0.254	1.038	0.933	0.214	0.167	0	2.699
10	BCbox-2	0.5	0.6	0.395	0.467	0.169	0.189	0	0.107	0	0.111	2.538
11	CBFb-2	0	0.1	0.296	0.28	0.085	0.094	0.667	0.321	0.333	0	2.177
12	APOBEC-8	0	0.1	0.099	0	0	0.66	0	0.536	0	0.111	1.506
13	Cul5-1	0	0	0	0	0	0.189	0.4	0	0.5	0.111	1.2
14	NLIS	0	0	0.099	0.187	0.338	0.189	0	0.107	0	0.222	1.142
15	Cul5-2	0	0	0.099	0	0	0	0	0.214	0	0.111	0.424
16	BCbox-1	0	0	0	0	0	0	0	0	0.333	0	0.333
**(b) MLP**												
**Rank**	**Variable**	**pos1**	**pos2**	**pos3**	**pos4**	**pos5**	**pos6**	**pos7**	**pos8**	**pos9**	**pos10**	**Total**
**1**	**APOBEC-2**	**6.7**	**0.36**	**0.495**	**0**	**0.207**	**0.074**	**0**	**0.075**	**0**	**0.136**	**8.047**
**2**	**APOBEC-3**	**0**	**4.05**	**0.825**	**0.692**	**0.276**	**0.147**	**0.17**	**0**	**0.148**	**0**	**6.308**
**3**	**BCbox-3**	**2.1**	**0.54**	**0.247**	**0.385**	**0.345**	**0.147**	**0.255**	**0.375**	**0.222**	**0.045**	**4.662**
4	APOBEC-5	0	0.63	1.567	1.385	0.345	0.588	0.085	0	0	0.045	4.645
5	APOBEC-6	0	0.81	1.155	0.769	0.828	0.294	0.255	0.225	0.074	0.045	4.455
6	APOBEC-4	0	0.36	1.897	0.231	0.621	0.294	0.34	0.225	0.148	0	4.116
7	Cul5-3	0.5	0.9	0.495	0.846	0	0.368	0	0.525	0	0.045	3.679
8	CBFb-1	0	0	0.33	0.385	0.552	0.735	0.596	0.15	0.148	0	2.895
9	APOBEC-8	0	0.27	0.082	0.846	0.276	0.368	0.511	0.15	0.074	0.045	2.622
10	Cul5-2	0	0.54	0.082	0.154	0.621	0.294	0.255	0	0.444	0.227	2.618
11	APOBEC-7	0.1	0	0.165	0.462	0.69	0.515	0.085	0.15	0.222	0.045	2.434
12	BCbox-2	0.5	0.27	0.165	0.385	0.276	0.147	0.255	0.225	0.074	0	2.297
13	NLIS	0	0.09	0.165	0.154	0.345	0.441	0.34	0.075	0	0.136	1.747
14	CBFb-2	0.1	0.18	0.165	0.077	0.138	0.221	0.426	0.075	0.148	0.136	1.665
15	Cul5-1	0	0	0.165	0.077	0.138	0.294	0.34	0.3	0	0.091	1.405
16	BCbox-1	0	0	0	0.154	0.345	0.074	0.085	0.45	0.296	0	1.404
(**c**) **NB**												
**Rank**	**Variable**	**pos1**	**pos2**	**pos3**	**pos4**	**pos5**	**pos6**	**pos7**	**pos8**	**pos9**	**pos10**	**Total**
**1**	**APOBEC-2**	**10**	**0**	**0**	**0**	**0**	**0**	**0**	**0**	**0**	**0**	**10**
**2**	**APOBEC-4**	**0**	**1.8**	**0.851**	**0.612**	**1**	**0.317**	**0.651**	**0.143**	**0.065**	**0.111**	**5.551**
**3**	**APOBEC-3**	**0**	**0.09**	**2.043**	**1.4**	**0.923**	**0.397**	**0.186**	**0.071**	**0.065**	**0**	**5.174**
4	APOBEC-6	0	0	0.596	0.875	0.538	0.794	0.837	0.143	0.323	0.111	4.217
5	APOBEC-8	0	1.89	0.426	0.525	0.231	0.238	0.279	0.286	0.129	0	4.003
6	BCbox-2	0	2.52	0.426	0.088	0.154	0	0.093	0.286	0.065	0.222	3.852
7	APOBEC-5	0	0	0.255	1.05	0.538	0.952	0.465	0.286	0.129	0.111	3.787
8	BCbox-3	0	0.54	0.17	0.7	0.846	0.873	0.093	0.286	0.129	0	3.637
9	Cul5-3	0	1.35	0.596	0.175	0.231	0.317	0.093	0.214	0.065	0	3.041
10	APOBEC-7	0	0	0.085	0.525	0.538	0.397	0.465	0.214	0.129	0.222	2.576
11	CBFb-2	0	0	0.681	0.438	0.385	0.159	0	0.143	0.387	0	2.192
12	NLIS	0	0.81	0.17	0.438	0.231	0.238	0	0.071	0.129	0	2.087
13	CBFb-1	0	0	0.17	0.175	0.231	0	0.372	0.357	0.323	0.111	1.739
14	Cul5-2	0	0	1.362	0	0.077	0.079	0	0.214	0	0	1.732
15	Cul5-1	0	0	0.085	0	0.077	0.159	0.372	0.143	0.065	0	0.9
16	BCbox-1	0	0	0.085	0	0	0.079	0.093	0.143	0	0.111	0.511
(**d**) **SVMs**												
**Rank**	**Variable**	**pos1**	**pos2**	**pos3**	**pos4**	**pos5**	**pos6**	**pos7**	**pos8**	**pos9**	**pos10**	**Total**
**1**	**APOBEC-2**	**8.8**	**0.329**	**0.107**	**0**	**0**	**0**	**0**	**0**	**0**	**0**	**9.236**
**2**	**BCbox-3**	**0.1**	**2.854**	**0.96**	**1.242**	**0.941**	**0.645**	**0.19**	**0.4**	**0**	**0**	**7.332**
**3**	**APOBEC-3**	**0**	**2.854**	**0.853**	**1.242**	**0.471**	**0.484**	**0**	**0**	**0.25**	**0.167**	**6.32**
4	APOBEC-5	0	0	0.427	1.016	1.529	0.806	0.571	0.6	0.25	0.167	5.367
5	APOBEC-4	0	0.659	2.133	0.565	0.824	0.323	0.571	0	0	0	5.074
6	APOBEC-7	0	0.659	1.067	0.452	0.118	0	0.762	0.4	0.25	0.167	3.873
7	APOBEC-6	0.1	0.11	0.64	0.339	0.706	0.484	0.19	0.4	0.5	0	3.469
8	BCbox-2	0.4	0.439	0.64	0.677	0.235	0.161	0.19	0.2	0	0.167	3.11
9	Cul5-3	0.4	0.439	0.64	0.226	0.471	0.323	0	0	0.5	0	2.998
10	APOBEC-8	0.1	0.22	0.32	0.339	0.118	0.645	0.571	0.2	0	0	2.512
11	NLIS	0.1	0.22	0.107	0.226	0.118	0.484	0.571	0.2	0	0	2.025
12	CBFb-2	0	0.11	0.107	0.452	0.353	0.323	0.19	0.2	0	0	1.734
13	CBFb-1	0	0	0	0.113	0.118	0.323	0.19	0.4	0.25	0.333	1.727
14	Cul5-2	0	0	0	0.113	0	0	0	0	0	0	0.113
15	BCbox-1	0	0.11	0	0	0	0	0	0	0	0	0.11
16	Cul5-1	0	0	0	0	0	0	0	0	0	0	0

**Table A3:** Relevance scores (*r*) in descending order per algorithm and variable considering the clinical endpoint **VLIni** using the MAREV-1 approach. The three variables with higher values are highlighted in bold.

(a) CART												
Rank	Variable	pos1	pos2	pos3	pos4	pos5	pos6	pos7	pos8	pos9	pos10	Total
**1**	**APOBEC-2**	**6.3**	**0.276**	**0.556**	**0**	**0**	**0**	**0.444**	**0**	**0**	**0**	**7.576**
**2**	**BCbox-1**	**0**	**5.235**	**0.667**	**0.75**	**0.13**	**0**	**0**	**0**	**0**	**0.25**	**7.032**
**3**	**APOBEC-3**	**0**	**0.827**	**1.778**	**1.625**	**0.783**	**0.167**	**0.148**	**0.2**	**0**	**0**	**5.527**
4	APOBEC-5	0	0.918	0.889	0.875	1.435	0.333	0.593	0	0	0	5.043
5	CBFb-2	1	0.551	1.333	0.75	0.13	0.167	0.296	0	0	0	4.228
6	APOBEC-4	0	0.643	1.556	0.5	0.522	0.667	0.296	0	0	0	4.183
7	APOBEC-7	0	0.276	0.667	0.375	0.652	0.5	0.296	0.2	0	0.25	3.216
8	APOBEC-6	0	0.092	0.222	0.375	0.783	0.833	0.296	0.4	0.2	0	3.201
9	BCbox-2	2.7	0	0.111	0.125	0	0	0.148	0	0	0	3.084
10	CBFb-1	0	0	0	0.125	0.522	1.167	0.296	0.2	0.2	0.25	2.76
11	Cul5-1	0	0	0	0	0.13	0.333	0.296	1	0.4	0	2.16
12	NLIS	0	0.092	0	0.375	0.261	0.5	0.148	0.6	0	0	1.976
13	Cul5-3	0	0.092	0.111	0.625	0.261	0	0.148	0.2	0.2	0	1.637
14	Cul5-2	0	0	0	0.125	0	0	0.296	0.2	0.6	0	1.221
15	APOBEC-8	0	0	0.111	0.125	0.261	0.167	0.148	0	0.4	0	1.212
16	BCbox-3	0	0	0	0.25	0.13	0.167	0.148	0	0	0.25	0.945
(**b**) **MLP**												
**Rank**	**Variable**	**pos1**	**pos2**	**pos3**	**pos4**	**pos5**	**pos6**	**pos7**	**pos8**	**pos9**	**pos10**	**Total**
**1**	**APOBEC-2**	**6.3**	**0.273**	**0.4**	**0.092**	**0**	**0.357**	**0.118**	**0.13**	**0.154**	**0.125**	**7.949**
**2**	**BCbox-1**	**0**	**5.182**	**0.5**	**0.276**	**0.689**	**0.476**	**0.118**	**0**	**0.154**	**0**	**7.394**
**3**	**APOBEC-5**	**0**	**0.545**	**1**	**0.276**	**1.082**	**0.952**	**0.471**	**0.261**	**0.154**	**0**	**4.741**
4	CBFb-2	1	0.818	1.7	0.368	0.098	0.357	0.118	0	0	0	4.46
5	APOBEC-3	0	1.182	0.8	1.105	0.59	0.238	0	0.13	0.308	0	4.353
6	CBFb-1	0	0	0.1	1.289	0.492	0.238	0.471	0.652	0.154	0.125	3.521
7	BCbox-2	2.7	0	0.3	0.092	0	0	0	0	0	0	3.092
8	Cul5-1	0	0	0.1	0.553	0.59	0.595	0.588	0.652	0	0	3.078
9	APOBEC-4	0	0.273	0.8	0.645	0.393	0	0.353	0.13	0.154	0	2.748
10	APOBEC-7	0	0.182	0.8	0.553	0.59	0.238	0.118	0.13	0	0	2.611
11	Cul5-2	0	0	0.1	0.092	0.295	0.833	0.471	0.261	0.308	0.125	2.485
12	NLIS	0	0	0.6	0.184	0.197	0.119	0.353	0.13	0.154	0.25	1.987
13	BCbox-3	0	0.091	0.1	0.737	0.197	0.238	0	0.13	0.308	0	1.801
14	APOBEC-8	0	0	0.3	0.092	0.492	0.119	0.353	0.13	0	0.25	1.736
15	APOBEC-6	0	0.182	0.3	0.368	0.098	0.119	0.235	0.13	0	0.125	1.558
16	Cul5-3	0	0.273	0.1	0.276	0.197	0.119	0.235	0.13	0.154	0	1.484
(**c**) **NB**												
**Rank**	**Variable**	**pos1**	**pos2**	**pos3**	**pos4**	**pos5**	**pos6**	**pos7**	**pos8**	**pos9**	**pos10**	**Total**
**1**	**APOBEC-2**	**10**	**0**	**0**	**0**	**0**	**0**	**0**	**0**	**0**	**0**	**10**
**2**	**Cul5-3**	**0**	**5.22**	**1.495**	**0.236**	**0.651**	**0.069**	**0**	**0.073**	**0**	**0.143**	**7.887**
**3**	**APOBEC-4**	**0**	**3.24**	**0.703**	**0.393**	**0.651**	**0.347**	**0.082**	**0.146**	**0**	**0.143**	**5.705**
4	CBFb-2	0	0	1.495	0.708	0.94	0.347	0.49	0.366	0.133	0	4.478
5	APOBEC-3	0	0	0.967	1.416	0.217	0.833	0.408	0.366	0.067	0	4.274
6	BCbox-1	0	0.36	1.319	0.787	0.578	0.208	0.327	0	0.067	0.143	3.788
7	BCbox-2	0	0	0.44	1.573	0.434	0.139	0.408	0.073	0.067	0.143	3.276
8	APOBEC-5	0	0	0.791	0.157	0.361	0.278	0.571	0.22	0.133	0.143	2.655
9	APOBEC-7	0	0	0.176	0.236	0.578	0.625	0.327	0.512	0.133	0	2.587
10	CBFb-1	0	0	0.088	0.157	0.578	0.833	0.163	0.293	0.4	0	2.513
11	NLIS	0	0.18	0.088	0.393	0.361	0.486	0.163	0.073	0.133	0.143	2.021
12	APOBEC-6	0	0	0.352	0.236	0.361	0.208	0.245	0.439	0	0	1.841
13	BCbox-3	0	0	0.088	0.157	0	0.347	0.245	0.293	0.2	0	1.33
14	APOBEC-8	0	0	0	0.236	0.072	0.069	0.408	0.073	0.333	0	1.192
15	Cul5-1	0	0	0	0.157	0.145	0.139	0.082	0.073	0.267	0	0.862
16	Cul5-2	0	0	0	0.157	0.072	0.069	0.082	0	0.067	0.143	0.59
**(d) SVMs**												
**Rank**	**Variable**	**pos1**	**pos2**	**pos3**	**pos4**	**pos5**	**pos6**	**pos7**	**pos8**	**pos9**	**pos10**	**Total**
**1**	**APOBEC-2**	**5.8**	**1.299**	**1.129**	**0.339**	**0**	**0.179**	**0**	**0**	**0**	**0**	**8.746**
**2**	**BCbox-2**	**2.1**	**1.485**	**1.6**	**0.339**	**0.15**	**0.179**	**0**	**0.167**	**0**	**0**	**6.018**
**3**	**APOBEC-3**	**0.2**	**1.206**	**1.318**	**1.581**	**0.6**	**0.357**	**0.462**	**0**	**0**	**0**	**5.723**
4	CBFb-2	0.3	2.876	1.035	0.565	0.15	0.179	0	0	0	0	5.105
5	APOBEC-5	0	0.093	0.188	0.903	1.05	0.357	0.615	0.667	0.364	0	4.237
6	APOBEC-7	0.4	0.186	0.565	0.226	1.8	0.179	0	0.333	0.364	0	4.052
7	BCbox-3	0.3	0.742	0.282	0.339	0	0.893	0.308	0	0.182	0.5	3.546
8	APOBEC-4	0.6	0.371	0.471	0.903	0.15	0.536	0.308	0	0.182	0	3.52
9	NLIS	0.1	0.186	0.376	0.113	0.45	0.536	0.923	0.5	0	0	3.184
10	Cul5-3	0.2	0.278	0.659	0.452	0.45	0.179	0.154	0.167	0.182	0	2.72
11	APOBEC-8	0	0.186	0	0.339	0.6	0.714	0.154	0.333	0.364	0	2.689
12	APOBEC-6	0	0	0.282	0.452	0.45	0.357	0.308	0	0.182	0	2.031
13	CBFb-1	0	0	0	0.113	0.15	0.357	0.154	0.5	0.182	0	1.456
14	BCbox-1	0	0.093	0.094	0.226	0	0	0.615	0.333	0	0	1.361
15	Cul5-1	0	0	0	0.113	0	0	0	0	0	0.5	0.613
16	Cul5-2	0	0	0	0	0	0	0	0	0	0	0

**Table A4:** Relevance scores (*r*) in descending order per algorithm and variable considering the clinical endpoint **VLHist** using the MAREV-1 approach. The three variables with higher values are highlighted in bold.

(a) CART												
Rank	Variable	pos1	pos2	pos3	pos4	pos5	pos6	pos7	pos8	pos9	pos10	Total
**1**	**NLIS**	**9.9**	**0**	**0**	**0**	**0.158**	**0**	**0**	**0**	**0**	**0**	**10.058**
**2**	**APOBEC-3**	**0**	**5.091**	**0.427**	**0.28**	**0**	**0**	**0.2**	**0**	**0**	**0**	**5.998**
**3**	**APOBEC-5**	**0**	**0.909**	**4.16**	**0.28**	**0**	**0**	**0.2**	**0**	**0**	**0**	**5.549**
4	APOBEC-2	0.1	2	0.747	0.7	0.474	0	0.4	0.5	0.4	0	5.32
5	CBFb-1	0	0	0.64	1.12	1.263	0.833	0.4	0	0	0	4.256
6	BCbox-1	0	0.636	0.853	0.42	0.632	0.333	0.4	0.5	0.4	0	4.175
7	APOBEC-8	0	0	0.213	1.82	0.632	0.5	0.8	0	0	0	3.965
8	APOBEC-7	0	0.182	0.107	0.42	0.632	0.5	0.8	0.25	0.4	0	3.29
9	Cul5-1	0	0	0.213	0.42	0.474	0.833	0.4	0.75	0	0	3.09
10	APOBEC-6	0	0	0.107	1.12	0.789	0	0	0.5	0	0	2.516
11	APOBEC-4	0	0.091	0.107	0.28	0	0.333	0.2	0.25	0	0.5	1.761
12	BCbox-3	0	0.091	0.32	0	0.158	1	0	0	0	0	1.569
13	CBFb-2	0	0	0.107	0.14	0.158	0.333	0	0	0	0.5	1.238
14	Cul5-2	0	0	0	0	0.158	0.167	0.2	0.25	0.4	0	1.175
15	BCbox-2	0	0	0	0	0.158	0.167	0	0	0.4	0	0.725
16	Cul5-3	0	0	0	0	0.316	0	0	0	0	0	0.316
(**b**) **MLP**												
**Rank**	**Variable**	**pos1**	**pos2**	**pos3**	**pos4**	**pos5**	**pos6**	**pos7**	**pos8**	**pos9**	**pos10**	**Total**
**1**	**NLIS**	**9.9**	**0**	**0**	**0**	**0**	**0**	**0**	**0**	**0**	**0**	**9.9**
**2**	**BCbox-1**	**0**	**3.33**	**0.791**	**1.28**	**0.312**	**0.137**	**0.123**	**0.45**	**0.16**	**0.053**	**6.636**
**3**	**CBFb-1**	**0**	**0**	**1.67**	**0.768**	**1.169**	**0.753**	**0.308**	**0.225**	**0**	**0**	**4.894**
4	APOBEC-5	0	0.81	1.319	1.11	0.39	0.479	0.185	0.075	0.4	0.053	4.82
5	APOBEC-3	0	2.43	0.44	0.683	0.234	0	0.246	0.075	0.16	0.053	4.32
6	Cul5-1	0	0	0.264	0.427	1.169	1.096	0.8	0.375	0.08	0.105	4.316
7	APOBEC-2	0.1	1.62	0.615	0.341	0.156	0.205	0.431	0.15	0.16	0.158	3.937
8	Cul5-2	0	0	0	0.256	1.325	0.753	0.862	0.375	0.16	0.105	3.836
9	APOBEC-8	0	0	0.44	0.854	0.39	0.137	0.492	0.45	0.32	0.053	3.135
10	APOBEC-7	0	0.09	1.319	0.427	0.078	0.205	0.185	0.225	0.16	0.053	2.741
11	APOBEC-4	0	0.36	0.527	0	0	0.274	0	0	0	0.158	1.319
12	APOBEC-6	0	0	0.352	0.085	0.312	0.205	0.062	0.15	0.08	0.053	1.298
13	BCbox-3	0	0.36	0	0.171	0.078	0.274	0.123	0.15	0.08	0.053	1.288
14	CBFb-2	0	0	0.264	0.171	0.234	0.137	0.062	0.15	0.08	0.053	1.149
15	Cul5-3	0	0	0	0.256	0.156	0.137	0.123	0.075	0.08	0.053	0.88
16	BCbox-2	0	0	0	0.171	0	0.205	0	0.075	0.08	0	0.531
**(c) NB**												
**Rank**	**Variable**	**pos1**	**pos2**	**pos3**	**pos4**	**pos5**	**pos6**	**pos7**	**pos8**	**pos9**	**pos10**	**Total**
**1**	**APOBEC-2**	**10**	**0**	**0**	**0**	**0**	**0**	**0**	**0**	**0**	**0**	**10**
**2**	**Cul5-3**	**0**	**1.71**	**3.2**	**0.794**	**0.174**	**0.574**	**0.073**	**0.077**	**0**	**0**	**6.601**
**3**	**NLIS**	**0**	**3.78**	**1.04**	**1.01**	**0.087**	**0.082**	**0.145**	**0.154**	**0.125**	**0**	**6.424**
4	BCbox-1	0	0.54	1.12	1.155	1.13	0.656	0.218	0.231	0.125	0	5.175
5	APOBEC-8	0	2.16	0.32	0.361	0.435	0.41	0.582	0.231	0	0	4.498
6	APOBEC-3	0	0.09	0.24	1.227	0.783	0.41	0.727	0.308	0.125	0.222	4.131
7	APOBEC-5	0	0	0.32	0.289	0.87	0.656	0.291	0.077	0.375	0	2.877
8	CBFb-1	0	0	0.16	0.433	0.435	0.656	0.364	0.308	0.125	0.111	2.591
9	APOBEC-7	0	0.09	0.08	0.505	0.261	0.41	0.218	0.385	0.25	0.222	2.421
10	BCbox-3	0	0	0.24	0.433	0.696	0.164	0.291	0	0.25	0	2.073
11	CBFb-2	0	0	0.56	0.289	0.174	0.41	0.145	0.231	0.125	0.111	2.045
12	BCbox-2	0	0.45	0.16	0.361	0.087	0.246	0.218	0.231	0	0	1.753
13	APOBEC-4	0	0.18	0.32	0	0.174	0.246	0.436	0	0.125	0	1.481
14	APOBEC-6	0	0	0.16	0.144	0.348	0	0.145	0.308	0.125	0.111	1.341
15	Cul5-1	0	0	0.08	0	0	0	0.145	0.231	0.25	0.111	0.817
16	Cul5-2	0	0	0	0	0.348	0.082	0	0.231	0	0.111	0.772
(**d**) **SVMs**												
**Rank**	**Variable**	**pos1**	**pos2**	**pos3**	**pos4**	**pos5**	**pos6**	**pos7**	**pos8**	**pos9**	**pos10**	**Total**
**1**	**NLIS**	**9.2**	**0.45**	**0.188**	**0.106**	**0**	**0**	**0**	**0**	**0**	**0**	**9.944**
**2**	**BCbox-3**	**0.1**	**3.51**	**0.941**	**0.636**	**0.8**	**0.568**	**0.2**	**0.097**	**0.2**	**0**	**7.052**
**3**	**APOBEC-2**	**0.3**	**1.8**	**1.976**	**0.955**	**0.533**	**0.227**	**0.1**	**0**	**0**	**0**	**5.892**
4	APOBEC-4	0	0.9	1.6	1.379	0.4	0.568	0.1	0.194	0.2	0	5.341
5	APOBEC-3	0	0.27	1.035	1.273	0.533	0.455	0.6	0.484	0	0	4.65
6	BCbox-1	0.2	0.27	0.376	0.742	0.133	1.023	0.7	0.387	0.6	0	4.432
7	APOBEC-8	0	0.09	0.471	0.424	1.333	0.227	0.3	0.194	0	0.556	3.595
8	APOBEC-5	0	0	0.282	0.636	0.667	0.455	0.5	0.29	0.2	0.222	3.252
9	BCbox-2	0	0.9	0.094	0.424	0.267	0.341	0.4	0.194	0	0	2.619
10	APOBEC-6	0	0	0.094	0.106	0.267	0.455	0.3	0.387	0.4	0	2.008
11	CBFb-2	0.2	0.63	0.376	0.106	0.133	0	0.1	0.097	0.2	0	1.843
12	Cul5-3	0	0	0.565	0.106	0.133	0.114	0.4	0.29	0	0	1.608
13	APOBEC-7	0	0.18	0	0.106	0.533	0.227	0.2	0.097	0	0	1.343
14	CBFb-1	0	0	0	0	0.267	0.114	0.1	0.097	0.2	0.111	0.888
15	Cul5-2	0	0	0	0	0	0.114	0	0.194	0	0	0.307
16	Cul5-1	0	0	0	0	0	0.114	0	0	0	0.111	0.225

Table A5 shows the results from the fourth step of the proposed methodology, see Section 3.5.

**Table A5:** Assessment on the variables considering the clinical endpoints using the MAREV-1 approach. The variables with values surpassing the calculated threshold are highlighted in bold.

CD4Ini						
Rank	Variable	CART	MLP	NB	SVMs	Total
**1**	**BCbox-3**	**8.282**	**8.136**	**7.906**	**8.585**	**32.909**
**2**	**APOBEC-3**	**5.782**	**4.406**	**10**	**5.64**	**25.828**
**3**	**APOBEC-5**	**6.982**	**6.516**	**5.673**	**5.607**	**24.778**
**4**	**APOBEC-2**	**5.504**	**4.572**	**3.53**	**6.631**	**20.237**
5	BCbox-2	3.511	3.78	4.717	6.455	18.463
6	APOBEC-6	6.038	5.059	2.749	3.335	17.181
7	APOBEC-4	2.33	3.283	2.7	5.083	13.396
8	Cul5-3	4.254	3.06	3.276	2.478	13.068
9	CBFb-1	3.031	3.114	2.596	2.875	11.616
10	APOBEC-7	1.449	1.761	2.278	3.471	8.959
11	APOBEC-8	2.917	1.826	1.715	0.562	7.02
12	NLIS	0.968	1.533	2.778	1.555	6.834
13	CBFb-2	1.633	1.939	1.513	1.387	6.472
14	Cul5-1	0.791	3.292	0.764	0.587	5.434
15	Cul5-2	0.566	1.39	2.21	0.672	4.838
16	BCbox-1	0.963	1.334	0.596	0.075	2.968
**CD4Hist**						
**Rank**	**Variable**	**CART**	**MLP**	**NB**	**SVMs**	**Total**
**1**	**APOBEC-2**	**8.393**	**8.047**	**10**	**9.236**	**35.676**
**2**	**APOBEC-3**	**6.767**	**6.308**	**5.174**	**6.32**	**24.569**
**3**	**APOBEC-5**	**5.467**	**4.662**	**3.637**	**7.332**	**21.098**
4	BCbox-3	5.359	4.116	5.551	5.074	20.1
5	APOBEC-4	5.958	4.645	3.787	5.367	19.757
6	Cul5-3	4.006	4.455	4.217	3.469	16.147
7	APOBEC-6	4.167	3.679	3.041	2.998	13.885
8	APOBEC-7	2.538	2.297	3.852	3.11	11.797
9	CBFb-1	2.865	2.434	2.576	3.873	11.748
10	BCbox-2	1.506	2.622	4.003	2.512	10.643
11	CBFb-2	2.699	2.895	1.739	1.727	9.06
12	APOBEC-8	2.177	1.665	2.192	1.734	7.768
13	Cul5-1	1.142	1.747	2.087	2.025	7.001
14	NLIS	0.424	2.618	1.732	0.113	4.887
15	Cul5-2	1.2	1.405	0.9	0	3.505
16	BCbox-1	0.333	1.404	0.511	0.11	2.358
**VLIni**						
**Rank**	**Variable**	**CART**	**MLP**	**NB**	**SVMs**	**Total**
**1**	**APOBEC-2**	**7.576**	**7.949**	**10**	**8.746**	**34.271**
**2**	**BCbox-1**	**5.527**	**4.353**	**4.274**	**5.723**	**19.877**
**3**	**APOBEC-3**	**7.032**	**7.394**	**3.788**	**1.361**	**19.575**
4	APOBEC-5	4.228	4.46	4.478	5.105	18.271
5	CBFb-2	5.043	4.741	2.655	4.237	16.676
6	APOBEC-4	4.183	2.748	5.705	3.52	16.156
7	APOBEC-7	3.084	3.092	3.276	6.018	15.47
8	APOBEC-6	1.637	1.484	7.887	2.72	13.728
9	BCbox-2	3.216	2.611	2.587	4.052	12.466
10	CBFb-1	2.76	3.521	2.513	1.456	10.25
11	Cul5-1	1.976	1.987	2.021	3.184	9.168
12	NLIS	3.201	1.558	1.841	2.031	8.631
13	Cul5-3	0.945	1.801	1.33	3.546	7.622
14	Cul5-2	1.212	1.736	1.192	2.689	6.829
15	APOBEC-8	2.16	3.078	0.862	0.613	6.713
16	BCbox-3	1.221	2.485	0.59	0	4.296
**VLHist**						
**Rank**	**Variable**	**CART**	**MLP**	**NB**	**SVMs**	**Total**
**1**	**NLIS**	**10.058**	**9.9**	**6.424**	**9.944**	**36.326**
**2**	**APOBEC-3**	**5.32**	**3.937**	**10**	**5.892**	**25.149**
**3**	**APOBEC-5**	**4.175**	**6.636**	**5.175**	**4.432**	**20.418**
4	APOBEC-2	5.998	4.32	4.131	4.65	19.099
5	CBFb-1	5.549	4.82	2.877	3.252	16.498
6	BCbox-1	3.965	3.135	4.498	3.595	15.193
7	APOBEC-8	4.256	4.894	2.591	0.888	12.629
8	APOBEC-7	1.569	1.288	2.073	7.052	11.982
9	Cul5-1	1.761	1.319	1.481	5.341	9.902
10	APOBEC-6	3.29	2.741	2.421	1.343	9.795
11	APOBEC-4	0.316	0.88	6.601	1.608	9.405
12	BCbox-3	3.09	4.316	0.817	0.225	8.448
13	CBFb-2	2.516	1.298	1.341	2.008	7.163
14	Cul5-2	1.238	1.149	2.045	1.843	6.275
15	BCbox-2	1.175	3.836	0.772	0.307	6.09
16	Cul5-3	0.725	0.531	1.753	2.619	5.628

Table A6 shows the results from the fifth step of the proposed methodology, see Section 3.5.

**Table A6:** The most informative variables per clinical endpoint considering those surpassing a calculated threshold (relevance scores in boldface). *a*, Scores when considering the classifications results from all the combinations; *b*, Scores calculated using only the best classification performance per clinical endpoint and algorithm (see Table 2).

	*a* MAREV-1					*b* MAREV-2			
Variable	CD4Ini	CD4Hist	VLIni	VLHist		CD4Ini	CD4Hist	VLIni	VLHist
APOBEC-2	**20.237**	**35.676**	**34.271**	19.099		6.5	**10.0**	**10.0**	**8.75**
APOBEC-3	**25.828**	**24.569**	**19.575**	**25.149**		7.75	2.25	5.083	3.75
APOBEC-4	13.396	19.757	16.156	9.405		1.75	**8.0**	**8.0**	2.25
APOBEC-5	**24.778**	**21.098**	18.271	**20.418**		2.5	3.25	5.167	1.5
APOBEC-6	17.181	13.885	13.728	9.795		1.25	1.5	1.667	3.667
APOBEC-7	8.959	11.797	15.47	11.982		0	5.167	2.25	1.0
APOBEC-8	7.02	7.768	6.713	12.629		0	1.667	3.333	4.833
BCbox-1	2.968	2.358	**19.877**	15.193		3.0	1.5	**6.5**	**7.167**
BCbox-2	18.463	10.643	12.466	6.09		**8.5**	3.667	5.0	1.75
BCbox-3	**32.909**	20.1	4.296	8.448		**8.5**	**7.0**	0	3.583
CBFb-1	11.616	11.748	10.25	16.498		0	0	2.0	1.667
CBFb-2	6.472	9.06	16.676	7.163		0	1.75	0	1.5
Cul5-1	5.434	7.001	9.168	9.902		0	0	0	1.333
Cul5-2	4.838	3.505	6.829	6.275		0	0	1.333	1.5
Cul5-3	13.068	16.147	7.622	5.628		5.25	4.25	1.667	2.25
NLIS	6.834	4.887	8.631	**36.326**		0	2.0	2.0	**8.5**
Threshold	20.2	20.25	19.18	19.85		8.187	6.328	6.454	5.326

Table A7 compares the findings of MAREV-1 and MAREV-2 with the previous results.

**Table A7:** Variables with the highest scores per clinical endpoint. a, Previous results [20]; b, Considering the MAREV-1 approach; c, Considering the MAREV-2 approach.

	*a Previous Results*			*b MAREV-1*			*c MAREV-2*	
Clinical Endpoint	Variable	Rank		Rank	Variable		Rank	Variable
CD4Ini	**BCbox-3**	1	=	1	**BCbox-3**	=	1	**BCbox-3**
	APOBEC-4	2		-	APOBEC-3		-	BCbox-2
	Cul-5	3		-	APOBEC-5			
				-	APOBEC-2			
CD4Hist	**APOBEC-2**	1	=	1	**APOBEC-2**	=	1	**APOBEC-2**
	**APOBEC-3**	2	=	2	**APOBEC-3**		-	APOBEC-4
				-	APOBEC-5		-	BCbox-3
VLIni	**APOBEC-2**	1	=	1	**APOBEC-2**	=	1	**APOBEC-2**
	**BCbox-1**	2	=	2	**BCbox-1**		-	APOBEC-4
	BCBox-2	3		-	APOBEC-3		-	BCbox-1
VLHist	**NLIS**	1	=	1	**NLIS**		-	APOBEC-2
	BCbox-1	2		-	APOBEC-3		-	NLIS
	APOBEC-2	3		-	APOBEC-5		-	BCbox-1

## References

[1] UNAIDS (2020). Data 2020. https://www.unaids.org/en/resources/documents/2020/unaids-data.

[2] Clercq E.D. (2005). Emerging anti-HIV drugs. Expert Opin. Emerg. Drugs.

[3] Greene W.C., Debyser Z., Ikeda Y., Freed E.O., Stephens E., Yonemoto W., Buckheit R.W., Esté J.A., Cihlar T. (2008). Novel targets for HIV therapy. Antivir. Res..

[4] Eberle J., Gürtler L.G. (2012). HIV Types, Groups, Subtypes and Recombinant Forms: Errors in Replication, Selection Pressure and Quasispecies. Intervirology.

[5] Scarlata S., Carter C. (2003). Role of HIV-1 Gag domains in viral assembly. Biochim. Biophys. Acta (BBA) Biomembr..

[6] Coloccini R.S., Dilernia D., Ghiglione Y., Turk G., Laufer N., Rubio A., Socías M.E., Figueroa M.I., Sued O., Cahn P. (2014). Host Genetic Factors Associated with Symptomatic Primary HIV Infection and Disease Progression among Argentinean Seroconverters. PLoS ONE.

[7] Goila-Gaur R., Strebel K. (2008). HIV-1 Vif, APOBEC, and Intrinsic Immunity. Retrovirology.

[8] Romani B., Engelbrecht S., Glashoff R.H. (2009). Antiviral roles of APOBEC proteins against HIV-1 and suppression by Vif. Arch. Virol..

[9] Beam A.L., Motsinger-Reif A., Doyle J. (2014). Bayesian neural networks for detecting epistasis in genetic association studies. BMC Bioinform..

[10] Jiang R., Tang W., Wu X., Fu W. (2009). A random forest approach to the detection of epistatic interactions in case-control studies. BMC Bioinform..

[11] Ritchie M.D., White B.C., Parker J.S., Hahn L.W., Moore J.H. (2003). Optimization of neural network architecture using genetic programming improves detection and modeling of gene-gene interactions in studies of human diseases. BMC Bioinform..

[12] Motsinger-Reif A.A., Lee S.L., Mellick G., Ritchie M.D. (2006). GPNN: Power studies and applications of a neural network method for detecting gene-gene interactions in studies of human disease. BMC Bioinform..

[13] Motsinger A., Dudek S., Hahn L., Ritchie M.D. (2006). Comparison of Neural Network Optimization Approaches for Studies of Human Genetics. Appl. Evol. Comput..

[14] Motsinger-Reif A.A., Ritchie M.D. (2008). Neural networks for genetic epidemiology: Past, present, and future. BioData Min..

[15] Tong D.L., Schierz A.C. (2011). Hybrid genetic algorithm-neural network: Feature extraction for unpreprocessed microarray data. Artif. Intell. Med..

[16] Cuevas-Tello J.C., Hernández-Ramírez D., García-Sepúlveda C.A. (2013). Support vector machine algorithms in the search of KIR gene associations with disease. Comput. Biol. Med..

[17] Boutorh A., Guessoum A. (2016). Complex diseases SNP selection and classification by hybrid Association Rule Mining and Artificial Neural Network—based Evolutionary Algorithms. Eng. Appl. Artif. Intell..

[18] Oriol J.D.V., Vallejo E.E., Estrada K., Peña J.G.T., Initiative T.A.D.N. (2019). Benchmarking machine learning models for late-onset alzheimer’s disease prediction from genomic data. BMC Bioinform..

[19] Hardin J., Waddell M., Page C.D., Zhan F., Barlogie B., Shaughnessy J., Crowley J.J. (2004). Evaluation of Multiple Models to Distinguish Closely Related Forms of Disease Using DNA Microarray Data: An Application to Multiple Myeloma. Stat. Appl. Genet. Mol. Biol..

[20] Altamirano-Flores J.S., Guerra-Palomares S.E., Hernandez-Sanchez P.G., Ramirez-Garcialuna J.L., Arguello-Astorga J.R., Noyola D.E., Cuevas-Tello J.C., Garcia-Sepulveda C.A. (2020). Identification of HIV-1 Vif Protein Attributes Associated With CD4 T Cell Numbers and Viral Loads Using Artificial Intelligence Algorithms. IEEE Access.

[21] López V., Fernández A., García S., Palade V., Herrera F. (2013). An insight into classification with imbalanced data: Empirical results and current trends on using data intrinsic characteristics. Inf. Sci..

[22] Zieba M., Tomczak J.M. (2014). Boosted SVM with active learning strategy for imbalanced data. Soft Comput..

[23] Guerra-Palomares S.E., Hernandez-Sanchez P.G., Esparza-Pérez M.A., Arguello J.R., Noyola D.E., García-Sepúlveda C.A. (2015). Molecular Characterization of Mexican HIV-1 Vif Sequences. AIDS Res. Hum. Retroviruses.

[24] Govender S., Otwombe K., Essien T., Panchia R., de Bruyn G., Mohapi L., Gray G., Martinson N. (2014). CD4 counts and viral loads of newly diagnosed HIV-infected individuals: Implications for treatment as prevention. PLoS ONE.

[25] Lane P.C., Clarke D., Hender P. (2012). On developing robust models for favourability analysis: Model choice, feature sets and imbalanced data. Decis. Support Syst..

[26] Hastie T., Friedman J., Tisbshirani R. (2017). The Elements of Statistical Learning: Data Mining, Inference, and Prediction.

[27] Haixiang G., Yijing L., Shang J., Mingyun G., Yuanyue H., Bing G. (2017). Learning from class-imbalanced data: Review of methods and applications. Expert Syst. Appl..

[28] Ignizio J. (1991). An Introduction to Expert Systems.

[29] Breiman L., Friedman J.H., Olshen R.A., Stone C.J. (1984). Classification and Regression Trees.

[30] Pedregosa F., Varoquaux G., Gramfort A., Michel V., Thirion B., Grisel O., Blondel M., Prettenhofer P., Weiss R., Dubourg V. (2011). Scikit-learn: Machine Learning in Python. J. Mach. Learn. Res..

[31] Buitinck L., Louppe G., Blondel M., Pedregosa F., Mueller A., Grisel O., Niculae V., Prettenhofer P., Gramfort A., Grobler J. API design for machine learning software: Experiences from the scikit-learn project. Proceedings of the ECML PKDD Workshop: Languages for Data Mining and Machine Learning.

[32] Singh S., Gupta P. (2014). Comparative study ID3, CART and C4.5 decision tree algorithm: A survey. Int. J. Adv. Inf. Sci. Technol..

[33] Mitchell T. (1997). Machine Learning.

[34] Rosenblatt F. (1957). The Perceptron—A Perceiving and Recognizing Automaton.

[35] Hinton G.E. (1989). Connectionist learning procedures. Artif. Intell..

[36] Rumelhart D.E., Hinton G.E., Williams R. (1986). Learning representations by back-propagating errors. Nature.

[37] Bishop C.M., Hinton G.E. (1995). Neural Networks for Pattern Recognition.

[38] Rojas R. (1996). Neural Networks: A Systematic Introduction.

[39] Haykin S. (1999). Neural Networks: A Comprehensive Foundation.

[40] Widrow B., Hoff M. (1962). Associative Storage and Retrieval of Digital Information in Networks of Adaptive ‘Neurons’. Biol. Prototypes Synth. Syst..

[41] Byrd R., Peihuang L., Nocedal J. (1996). A Limited-Memory Algorithm for Bound-Constrained Optimization.

[42] Gunn S. (1998). Support Vector Machines for Classification and Regression.

[43] Shawe-Taylor J., Cristianini N. (2004). Kernel Methods for Pattern Analysis.

[44] Hall M., Frank E., Holmes G., Pfahringer B., Reutemann P., Witten I.H. (2009). The WEKA Data Mining Software: An Update. SIGKDD Explor.

[45] Simon J.H.M., Sheehy A.M., Carpenter E.A., Fouchier R.A.M., Malim M.H. (1999). Mutational Analysis of the Human Immunodeficiency Virus Type 1 Vif Protein. J. Virol..

[46] Chen G., He Z., Wang T., Xu R., Yu X.F. (2009). A Patch of Positively Charged Amino Acids Surrounding the Human Immunodeficiency Virus Type 1 Vif SLVx4Yx9Y Motif Influences Its Interaction with APOBEC3G. J. Virol..

